# Tfh2 and a subset of Tfh1 cells associate with antibody-mediated immunity to malaria

**DOI:** 10.1172/jci.insight.196828

**Published:** 2025-10-21

**Authors:** Megan S.F. Soon, Damian A. Oyong, Nicholas L. Dooley, Reena Mukhiya, Zuleima Pava, Dean W. Andrew, Jessica R. Loughland, James S. McCarthy, Jo-Anne Chan, James G. Beeson, Christian R. Engwerda, Ashraful Haque, Michelle J. Boyle

**Affiliations:** 1 Burnet Institute, Melbourne, Victoria, Australia.; 2 QIMR Berghofer, Herston, Queensland, Australia.; 3 Environment and Sciences, Griffith University, Queensland, Australia.; 4 Faculty of Medicine, Dentistry and Health Sciences, University of Melbourne, Victoria, Australia.; 5 Department of Microbiology and School of Translational Medicine, Monash University, Victoria, Australia.

**Keywords:** Immunology, Infectious disease, Malaria

## Abstract

High-affinity antibody production depends on CD4^+^ T follicular helper (Tfh) cells. In humans, peripheral blood Tfh cells are heterogenous, as evidenced by differential expression of the chemokine receptors CXCR3 and CCR6, which to date have served to classify 3 subsets, pTfh1, pTfh2, and pTfh17. Although pTfh1 responses dominate during blood-stage *Plasmodium* infections, a clear association with protective antibody responses remains to be described. We hypothesized that pTfh cells exhibit greater phenotypic and functional heterogeneity than described by CXCR3/CCR6 and that more nuanced pTfh subsets play distinct roles during *Plasmodium* infection. We mapped pTfh cell heterogeneity in healthy individuals prior to and during controlled human malaria infection (CHMI) using parallel single-cell RNA-Seq and VDJ-Seq. We uncovered 2 pTfh1 subsets or differential phenotypic states, distinguishable by CCR7 expression. Prior to infection, Tfh1-CCR7^–^ cells exhibited higher baseline expression of inflammatory cytokines and genes associated with cytotoxicity. Tfh1-CCR7^+^ cells had higher germinal center signatures. Indeed, during CHMI, Tfh1-CCR7^+^, Tfh1-CCR7^–^, and Tfh2 cells all clonally expanded and became activated. However, only Tfh1-CCR7^+^ and Tfh2 cells positively associated with protective antibody production. Hence, our data reveal further complexity among human Tfh cells and highlight 2 distinct subsets associated with antibody-mediated immunity to malaria.

## Abstract 2

We dissect cell heterogeneity in malaria infection with scRNAseq and identify two distinct Tfh cell phenotypes that associate with antibody mediated immunity in malaria.

## Graphical abstract

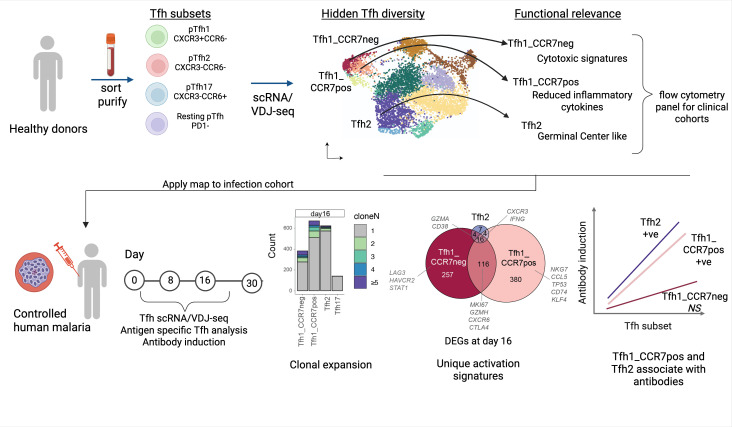

## Introduction

Antibodies and B cells induced by infection or vaccination are essential mediators of protection from most pathogens. This includes malaria, caused by *Plasmodium falciparum* infection, which remains one of the largest killers globally (1). While the introduction of 2 malaria vaccines, RTS,S/AS01 and R21/Matrix-M, bring new hope to malaria control, these vaccines only have modest, short-lived, and variable efficacy (2, 3). Antibody development is driven by T follicular helper (Tfh) CD4^+^ T cells, which function within the germinal center (GC) to support B cell activation. Cytokine- and receptor-mediated signals from Tfh cells drive B cell selection, support antibody class switching, and support differentiation into antibody-producing, long-lived plasma cells and memory B cells. As such, Tfh cells are attractive targets to boost naturally acquired or vaccine-mediated protection and longevity (4). To develop such therapies, an improved understanding of Tfh cells in malaria and specific disease contexts is required.

Tfh cells, identified by expression of CXC chemokine receptor 5 (CXCR5) and programmed cell death protein 1 (PD-1), exist in distinct subsets, which have specific and context-dependent roles in antibody induction. These Tfh subsets take on characteristics of CD4^+^ T cell helper cell lineages (5) and are commonly analyzed by CXCR3 and CCR6 expression to identify Tfh1 (CXCR3^+^CCR6^–^), Tfh2 (CXCR3^–^CCR6^–^), and Tfh17 (CXCR3^–^CCR6^+^) cells, both in the periphery and also in GC Tfh cells in secondary lymphoid tissues (6, 7). The development and activation of Tfh subsets may be underpinned by skewing of Tfh cells by the induced cytokine milieu, allowing Tfh cells to adopt specific roles to influence B cell development (5, 8). Indeed, defined Tfh cell subsets have differing abilities to activate naive or memory B cells and have been associated with antibody development in a pathogen- and vaccine-dependent manner. For example, Tfh2 cells have the highest capacity to activate naive B cells, and peripheral (p)Tfh2 cells have been associated with broadly neutralizing antibodies in HIV infection (9). On the other hand, Tfh1 cells robustly activate memory B cells, and the activation of pTfh1 cells is consistently associated with antibodies induced by influenza vaccination (10, 11). Thus, strategies to target Tfh cells to boost antibody development require a context-specific understanding of specialized Tfh subsets.

In malaria, progress has been made to define roles of Tfh cells in antibody development in infection and vaccination (12). Studies by our team have shown that activation of pTfh2 cells was associated with antibody development during controlled human malaria infection (CHMI) with *P*. *falciparum* (13). Similarly, others have shown that pTfh2 cells are associated with induction of antibodies by approved and experimental malaria vaccines (14–16). These associations are observed despite the overall response during malaria being dominated by pTfh1 cells (13, 17, 18). Further, in Malian children, pTfh1 cell activation was not associated with antibody development following malaria treatment (18), and pTfh1 cells have been associated with induction of short-lived plasma cells that may hamper the GC (13, 19). As such, data suggest that strategies that can boost Tfh2 in preference to Tfh1 may improve malaria antibody development. However, recent studies have shown that these relationships are more nuanced, with Ugandan children who had the highest levels of antibodies having increased frequencies of activated pTfh2 and also pTfh1 cells (20). Additionally, parasite-activated pTfh1 cells induced T-bet^+^ B cells with IgG3 expression (21), and IgG3 antibodies are consistently associated with protection from malaria (22).

We hypothesized that contradictory results may be in part due to limited approaches to identify Tfh subsets, and an improved understanding of Tfh heterogeneity would reveal protective subsets or phenotypic states associated with antibody development in malaria. In this study, we applied single-cell RNA-Seq (scRNA-Seq) and spectral flow cytometry to discover functionally relevant Tfh cell subsets/cell states that have differing roles in malaria antibody development.

## Results

### Analysis of Tfh cells with scRNA-Seq identifies 2 Tfh1 clusters with distinct transcriptional states.

**Figure 1:**
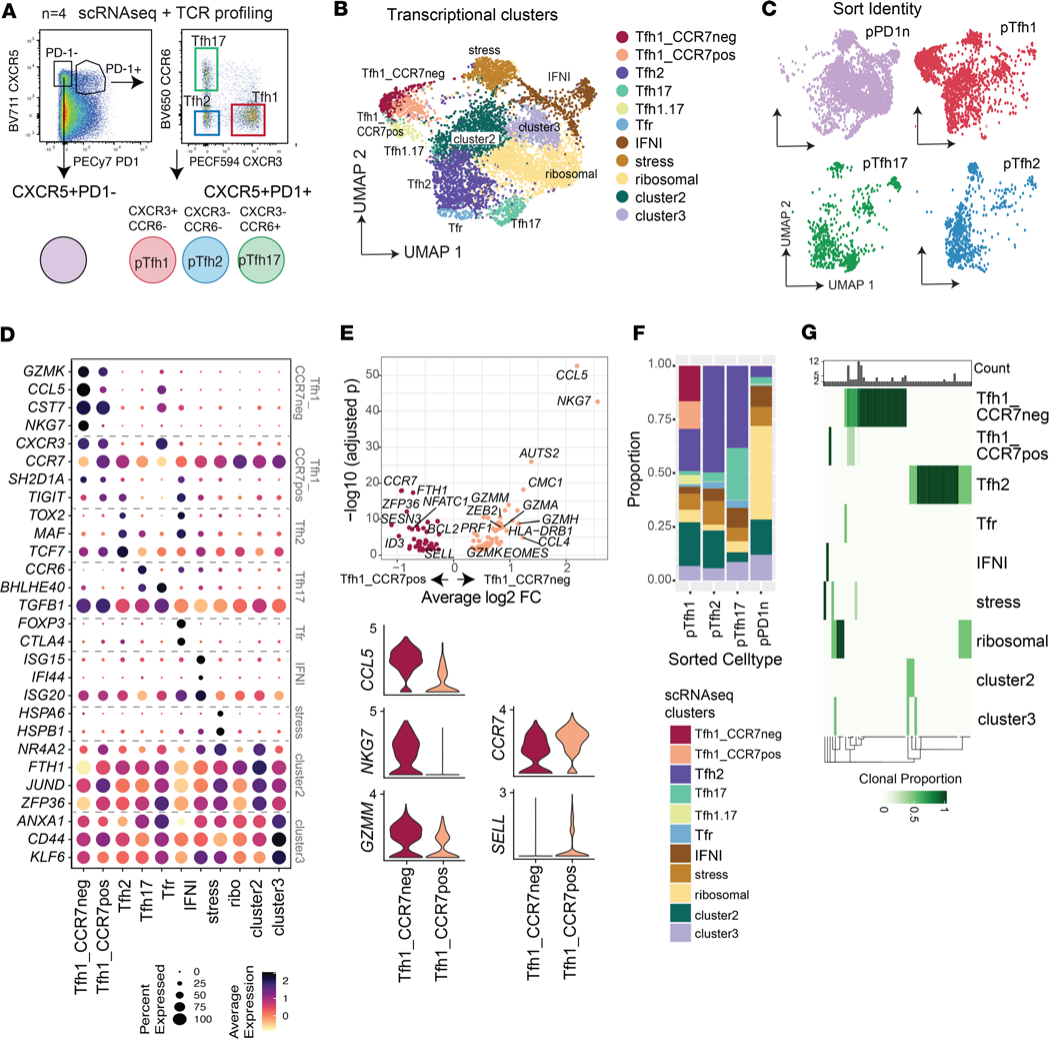
scRNA-Seq analysis of pTfh cells from healthy individuals. (**A**) Four populations were sorted from pTfh CD4^+^ cells from 4 healthy donors — 1) CXCR5^+^PD1^–^, 2) CXCR5^+^PD1^+^ pTfh1, 3) CXCR5^+^PD1^+^ pTfh2, and 4) CXCR5^+^PD1^+^ pTfh17. Populations were analyzed by 5′ droplet scRNA-Seq for gene expression and TCR sequencing. (**B**) Uniform manifold approximation and projection (UMAP) of data following integration by donor, with 11 transcriptional subsets indicated. IFNI, type I IFN; Tfh1.17, mixed Tfh1/Tfh17 signature. (**C**) UMAP of data depicting cell sorted identity. (**D**) Relative gene expression of genes used to annotate transcriptional clusters. (**E**) DEGs comparing Tfh1_CCR7neg and Tfh1_CCR7pos subsets. Average log2 FC and –log10 adjusted *P* values are shown (top panel). Average expression of key genes in Tfh1_CCR7neg and Tfh1_CCR7pos transcriptional clusters (bottom panels). Violin plots show complete data distribution of gene expression across all cells. (**F**) Relationship between cell sorted identity and transcriptional cluster identity in the dataset. (**G**) Fate sharing at the individual-clonotype level across transcriptional subsets. Expanded clonotypes (*n* ≥ 2) with only same fates (exist within the same subset) or different fates are shown. See also Supplemental Figures 1 and 2 and Supplemental Tables 1 and 2.

We hypothesized that hidden diversity of Tfh cells not captured in traditional analysis methods underpinned the unclear roles of Tfh subsets in antibody development in malaria. To dissect this Tfh diversity, we first performed scRNA-Seq on pTfh cells sorted from healthy donors (*n* = 4, sex 50% males, aged 28.5 [25.5–35], median [IQR] years). To understand the link between commonly identified Tfh subsets and potential hidden diversity, we sorted 3 Tfh subsets (pTfh1, pTfh2, and pTfh17 from CXCR5^+^PD1^+^ cells), based on classical definitions of CXCR3/CCR6 expression, alongside less functional or less activated CXCR5^+^PD1^–^ cells (Figure 1A and Supplemental Figure 1A; supplemental material available online with this article; https://doi.org/10.1172/jci.insight.196828DS1). After quality control (QC), 7,302 cells were analyzed with high-quality cells captured from each donor and phenotypic subset (pTfh1 *n* = 2,089, pTfh2 *n* = 717, pTfh17 *n* = 744, PD-1 *n* = 2,839, Supplemental Figure 1B). Following data integration (Supplemental Figure 1C), we identified 11 Tfh cell clusters using a combination of unsupervised and semi-supervised methods (Figure 1, B–D, and Supplemental Table 1). Clusters overlapped across Tfh phenotypically sorted subsets (Figure 1C), despite enrichment of gene expression of phenotypic marker genes used for sorting (Supplemental Figure 1D). Strikingly, 2 transcriptionally distinct Tfh1 clusters were identified, both with relatively high expression of *CXCR3* and *CST7* (annotated Tfh1-CCR7^–^ and Tfh1-CCR7^+^ based on subsequent analysis). A Tfh2 cluster was identified with high expression of genes associated with GC Tfh cells, including *TOX2* (23, 24), *MAF* (25, 26), and *TCF7*. The signature of the Tfh2 cluster mapped to tonsil GC Tfh cells in a publicly available data set (27) (Supplemental Figure 2A), confirming transcriptional relationships between Tfh2 cells and GC Tfh cells reported previously (9). A Tfh17 cluster was annotated based on high expression of genes associated with Th17 cells, *CCR6*, *BHLHE40*, and *TGFB1* (28–30). Most cells within these Tfh1/Tfh2 and Tfh17 transcriptional clusters were sorted from the PD1^+^ cells (Figure 1, B and C). Other transcriptional clusters included cells enriched in type I IFN genes (annotated as IFNI), a cluster enriched with heat shock proteins (annotated stress), another cluster enriched with ribosomal genes (annotated ribosomal), and 2 clusters without clear marker genes (cluster 2, possibly quiescent cells, with upregulation of genes *BTG1* and *ZFP36L2*, and cluster 3, which only had limited upregulated genes, including annexin family members *ANXA1*, *ANXA2*, *S100A10*) (Figure 1D). Analysis of key T helper genes also identified a small cluster of cells positioned close to the Tfh1 clusters that expressed a mixed Tfh1/Tfh17 signature, which were annotated as Tfh1.17 (Figure 1, B and D). Additionally, another cluster of cells expressing *FOXP3* and Treg signature genes, including *CTLA4*, was identified manually and annotated as Tfr (Figure 1D). The remaining Tfh cell clusters expressed diverse levels of markers associated with various aspects of Tfh or CD4^+^ T cell development (Supplemental Figure 2B).

To compare the 2 Tfh1 clusters, we identified significant differentially expressed genes (DEGs) between the 2 clusters (Supplemental Table 2). A total of 66 DEGs were significantly upregulated in the Tfh1-CCR7^–^ cluster, and 41 DEGs were significantly upregulated in the Tfh1-CCR7^+^ cluster (Figure 1E). Genes upregulated in Tfh1-CCR7^–^ cells included multiple genes associated with cytotoxicity and inflammatory responses, including *GZMK*, *GZMM*, *GZMA*, *CCL5*, *CCL4*, and *NKG7* (Figure 1, D and E; Supplemental Figure 2B; and Supplemental Table 2). The signature of the Tfh1-CCR7^–^ cluster overlaps with cytotoxic Tfh cells previously identified in mouse models, including the increased expression of *EOMES* (31), and in a recent small analysis of human peripheral and tonsil Tfh cells (32) (Supplemental Figure 2B). Granzyme K–expressing pTfh cells have also recently been identified in type I skewed response in mice and in human CMV (33). In contrast, the Tfh1-CCR7^+^ cluster had reduced expression of cytotoxic markers and had increased expression of *CCR7* and *SELL*. Based on these data, the 2 clusters were annotated as Tfh1-CCR7^–^ and Tfh1-CCR7^+^. Expression of CCR7 along with PD-1 has previously been used to differentiate Tfh subsets within CXCR5^+^ cells in both mice and humans, with CCR7^lo^PD1^hi^ described as effector cells and CCR7^hi^PD1^lo^ described as resting cells (34). However, here both Tfh1-CCR7^–^ and Tfh1-CCR7^+^ subsets largely existed in clusters that were from the PD1^+^ sorted populations, thus identifying an unappreciated gradient of CCR7 expression within PD1^+^ cells (Figure 1, C and F). Also upregulated in Tfh1-CCR7^+^ cells was *NFATC1* (also known as *NFAT2*, expressing nuclear factor of activated T cells 2 [NFAT2] protein) which positively regulates GC Tfh differentiation in mice during acute lymphocytic choriomeningitis virus (LCMV) (35). Pathway analysis of Tfh1-CCR7^+^ and Tfh1-CCR7^–^ cluster marker genes (identified in comparison with all other Tfh clusters) highlighted the shared and divergent transcriptional signatures of these cells (Supplemental Figure 2C). For example, both subsets were enriched for Th1 pathway and interferon gamma signalling; however, this enrichment was comparatively greater for Tfh1-CCR7^–^. However, only the Tfh1-CCR7^+^ cluster was enriched in Th2 pathway and ICOS-ICOSL signalling in T helper cells, suggesting potential functional differences between the 2 clusters (Supplemental Figure 1C). Together with the distinct expression of cytotoxic markers, data suggest Tfh1 cells exist in 2 distinct and functionally relevant subsets or cell states.

We analyzed the degree of overlap between Tfh transcriptional clusters and sorted cell phenotypes. Tfh1-CCR7^–^, Tfh1-CCR7^+^, Tfh2, and Tfh17 clusters were mainly found in cells expressing *PDCD1* (expressing PD-1). Further, *CXCR3* and *CCR6* expression overlapped with the pTfh1 and Tfh17 sorted populations, respectively, suggesting some overlap between transcriptional clusters and those identified via CXCR3 and CCR6 expression (Figure 1C and Supplemental Figure 1C). Nearly all cells found within both Tfh1 transcriptional clusters were from the pTfh1 sorted cell population. However, the sorted pTfh1 population also contained cells from all other transcriptional clusters. Similarly, the phenotypic pTfh2 population was dominated by the Tfh2 transcriptional cluster but also contained cells with diverse transcriptional annotations (Figure 1F and Supplemental Figure 2D). This pattern was also seen for the phenotypic pTfh17 population. The PD-1 phenotypic cells contained cells of all clusters but were dominated by IFNI, stress, ribosomal, cluster 2, and cluster 3 cells.

To understand the clonal sharing between clusters, we analyzed TCR sequences. Recent studies have suggested clonal fidelity across T cell clusters (36), and analysis of CXCR3^+^ Tfh cells in the blood and tonsil has shown minimal clonal sharing between CXCR3^+^ and CXCR3^–^ cells (7). As such we hypothesized that shared clones across transcriptional clusters would suggest clusters could be different cell states of otherwise related subsets, while clonal fidelity would suggest clusters were distinct subsets. TCR sequences with complete TRA/TRB genes were captured for 3,998 (55.6%) cells. While the majority of TRA/TRB cells were singletons (3,839 clones), clonal expansion was detected in 159 cells across 57 clones ranging in size from 2–20 clones. Clonal expansion was largest within the Tfh1-CCR7^–^ subset (Supplemental Figure 2E). As expected, no clones were shared across individuals (Supplemental Figure 2F). Within each clonotype, 40/57 clonotypes (70.2%) were found in a single cluster. Clonal sharing was evident between Tfh1-CCR7^–^ and Tfh1-CCR7^+^ but not Tfh2 transcriptional clusters, while Tfh2 shared clones with ribosomal, cluster 2, and cluster 3 cells (Figure 1G). Together, these data show that while commonly used phenotypic markers CXCR3 and CCR6 can resolve some heterogeneity in pTfh cells, alternative distinct Tfh clusters, particularly within the pTfh1 compartment, may require additional phenotypic markers to dissect subsets or cell states of potential functional relevance.

### pTfh1 cells with distinct phenotypic and functional characteristics defined by CCR7.

**Figure 2:**
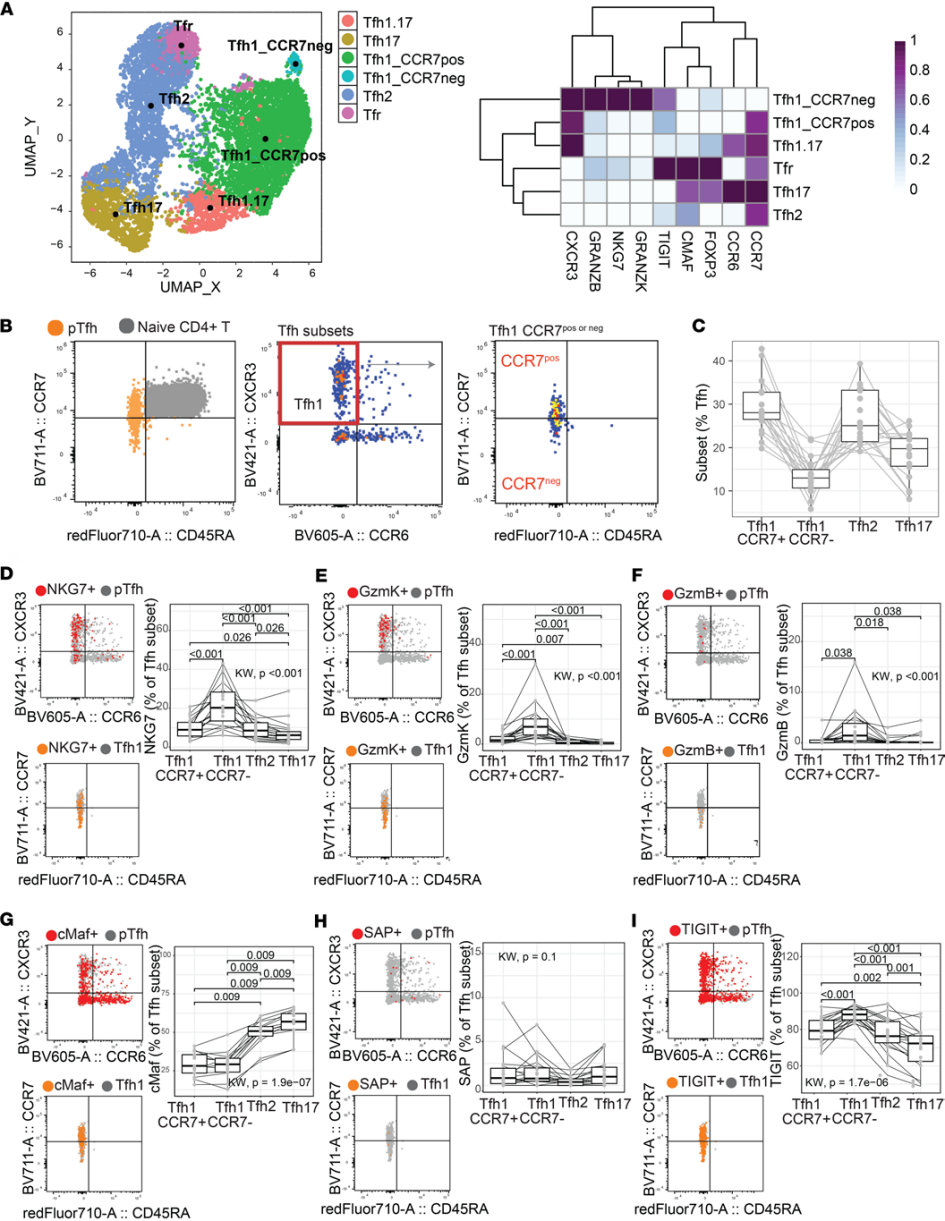
Phenotypic diversity of pTfh cell subsets based on CCR7 expression. A comprehensive spectral flow cytometry panel was used to analyze pTfh cells in healthy individuals (*n* = 19). (**A**) pTfh cells were identified by CXCR5 and PD-1 and analyzed with unbiased analysis of indicated markers. UMAP of a single experiment analyzing *n* = 7 individuals. Analysis performed 3 independent times. (**B**) Tfh cells were analyzed with manual gating. A gradient of CCR7 expression is detected within Tfh cells, and Tfh1-CCR7neg (CCR7^–^) and Tfh1-CCR7pos (CCR7^+^) subsets can be identified, along with Tfh2 and Tfh17 cells. (**C**) Proportion of Tfh subsets within total Tfh population. (**D**–**I**) Expression of cytotoxic markers NKG7 (**D**), granzyme K (**E**), and granzyme B (**F**) and transcriptional factors cMaf (**G**), SAP (**H**), and TIGIT (**I**) were analyzed within Tfh cell subsets. Flow plots are a representative individual with marker expression overlaid against CXCR3 and CCR6 or CCR7 and CD45RA. Box plots with median and IQR are indicated along with individuals. *P* is the paired Wilcoxon signed-rank test after adjustment with multiple-testing correction using Holm’s method, while the Kruskal-Wallis test is used for the global comparison. Only significant differences (*P* < 0.05) are shown. See also Supplemental Figures 3 and 4.

To assess if Tfh subsets/states could be identified phenotypically and develop an approach to identify these subsets/states in clinical cohorts, we designed a comprehensive phenotyping flow cytometry panel for pTfh cells, which included commonly assessed chemokine receptors and markers identified in our transcriptional data set or with reported roles in Tfh cell function or development (Supplemental Table 3). We first analyzed pTfh cells in healthy donors (*n* = 19, sex 83.3% males, aged 24 years [20.2–28.2], median [IQR] years). pTfh cells were identified based on CXCR5 and PD-1, then analyzed with unsupervised clustering considering CXCR3, CCR6, CCR7, NKG7, GrzmB, GrzmK, cMAF, TIGIT, and FoxP3. As seen in transcriptomic analysis, unbiased clustering consistently identified 2 clusters of pTfh1 cells based on CCR7 expression, where CCR7^–^ cells expressed high levels of NKG7, GrzmB, and GrzmK, and CCR7^+^ cells were negative for these markers (Figure 2A and Supplemental Figure 2, A–C). pTfh2, pTfh17, and pTfh1.17 cells could also be identified (Figure 2A). Additionally, FoxP3^+^ Tfr cells were identified and largely clustered within the pTfh2 cell subset, consistent with scRNA-Seq data (Figure 2A and Supplemental Figure 2, A–C).

To assess if analysis of a single marker, CCR7, together with CXCR3 and CCR6, could capture this underlying diversity in Tfh1 cells, we next applied manual gating approaches. Use of CCR7 as a distinguishing marker was chosen over NKG7 or GrzmB/K as surface-expressed proteins are more readily applied to large sample cohorts without the need for intracellular staining. We analyzed all non-naive CD4^+^ T cells, including effector memory reexpressing CD45RA (TEMRA, CCR7^+^CD45RA^–^), which were not analyzed within scRNA-Seq (Supplemental Figure 2D). However, consistent with prior reports (13, 37), only a very small proportion of Tfh were TEMRA cells (0.42% [0.275–0.695], Supplemental Figure 2E). Within pTfh cells (CXCR5^+^PD1^+^ [FoxP3^–^CD25^–^] CD4^+^ T cells) a gradient of CCR7 was detected, and Tfh1 cells could be divided into CCR7^+^ and CCR7^–^ (Figure 2B and Supplemental Figure 2D). Within total Tfh, Tfh1-CCR7^+^ and Tfh2 cells were the dominant subsets, followed by Tfh17 and Tfh1-CCR7^–^ cells (Figure 2C). Expression of cytotoxic markers NKG7, GrzmB, and GrzmK was significantly higher in Tfh1-CCR7^–^ compared with other subsets (Figure 2, D–F, and Supplemental Figure 2E). Within Tfh cells, we also quantified cMaf, which was upregulated transcriptionally in Tfh2 cell clusters, and SAP (*SH2D1A*) and TIGIT, which were increased transcriptionally in the Tfh1-CCR7^+^ cluster (Figure 2, G–I). cMaf and SAP have essential roles in GC Tfh cell development (38), and TIGIT is reported to have highest expression in GC Tfh and Tfh2 cells (9). cMaf expression was not different between Tfh1-CCR7^–/+^ cells, both of which had significantly lower expression compared with Tfh2 and Tfh17, consistent with previous reports (9) (Figure 2G). SAP expression was low across all subsets, was lowest in Tfh2, and was comparable between the 2 Tfh1 subsets (Figure 2H). Further, in contrast with previous reports, TIGIT expression was highest on Tfh1-CCR7^–^ cells compared with both Tfh1-CCR7^+^ and Tfh2 (Figure 2I). When considering expression of these markers as MFI in each Tfh subsets, patterns of expression were similar to expression as percentage of positive, except for granzyme B, which was comparable across subsets (Supplemental Figure 3, A–F). Thus, while CCR7 could differentiate populations of pTfh1 cells with distinct cytotoxic potential, how key transcriptional factors may influence pTfh subsets requires further investigation.

**Figure 3:**
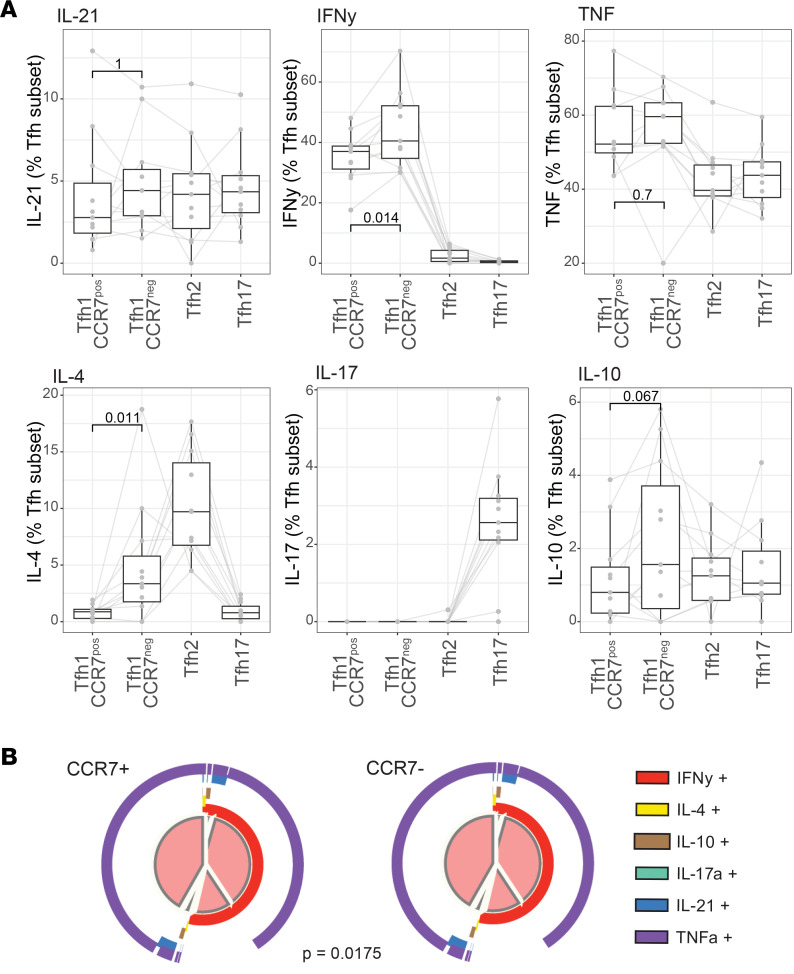
Functional diversity within pTfh cell subsets based on CCR7 expression. Cytokine production following PMA/Io stimulation was measured by intracellular staining in healthy individuals (*n* = 11). pTfh cell subsets were identified by CXCR3 and CCR6 expression as pTfh1 (CXCR3^+^CCR6^+^), pTfh2 (CXCR3^–^CCR6^–^), and pTfh17 (CXCR3^–^CCR6^+^), and pTfh1 cell subsets were identified as CCR7^pos^ and CCR7^neg^ cells. (**A**) IL-21, IFNG, TNF, IL-4, IL-17, and IL-10 expression within pTfh cell subsets. *P* is the paired Wilcoxon signed-rank test between pTfh1 cell subsets. (**B**) Overall cytokine production within pTfh1 CCR7^pos^ and CCR7^neg^ cells. *P* is permutation test in Simplified Presentation of Incredibly Complex Evaluations (SPICE). Box-and-whisker plots indicate first and third quartiles for hinges, median line, and lowest and highest values no further than 1.5 IQR from hinges. All individual data are represented by the gray dots and lines. See also Supplemental Figure 5.

To further analyze potential functional differences of Tfh1 cell subsets/states, cytokine production from Tfh cells was quantified following PMA/ionomycin (Io) stimulation in healthy donors (Supplemental Figure 4 and Supplemental Table 4, *n* = 11, sex 36.36% males, age 37.5 [32–40], median [IQR] years). As seen ex vivo, a gradient of CCR7 expression was detected within Tfh1 cell subsets (CXCR3^+^CCR6^–^) poststimulation (Supplemental Figure 4, A and B). Stimulation conditions were optimized to minimize changes in cell subsets, and there were no differences in the frequencies of Tfh cells, nor Tfh1-CCR7^+^ and Tfh1-CCR7^–^ subsets, and only a small difference in Tfh2 proportions in stimulated compared with unstimulated cultures (Supplemental Figure 4C). Following stimulation the proportion of IL-21–positive cells (the Tfh-related cytokine) was comparable across commonly identified Tfh1, Tfh2, and Tfh17 cell subsets, while the proportion of IFN-γ– and TNF-positive cells was higher in Tfh1, the proportion of IL-4–positive cells higher in Tfh2, and the proportion of IL-17–positive cells higher in Tfh17, as expected (Supplemental Figure 4, D and E). The regulatory cytokine IL-10 was detected at low levels in all subsets. When Tfh1 cells were analyzed based on CCR7 expression, there was no difference between Tfh1-CCR7^+^ and Tfh1-CCR7^–^ in proportion of IL-21– or TNF-positive cells; however, Tfh1-CCR7^–^ cells produced significantly higher IFN-γ and IL-4 and a trend toward increased IL-10 (Figure 3A). Increased production of IFN-γ is consistent with higher expression of inflammatory genes, including *CCL4* and *CCL5*, in Tfh1-CCR7^–^ cells transcriptionally. When the total cytokine composition was analyzed, there was a significant difference in the cytokine composition produced by Tfh1-CCR7^+^ compared with Tfh1-CCR7^–^ cells (Figure 3B). Given the importance of the milieu Tfh cytokine production in B cell responses (5), data suggest that Tfh1-CCR7^+/–^ subsets may have nuanced roles in antibody induction.

### Expansion of pTfh1 and pTfh2 cell subsets in human malaria infection.

**Figure 4:**
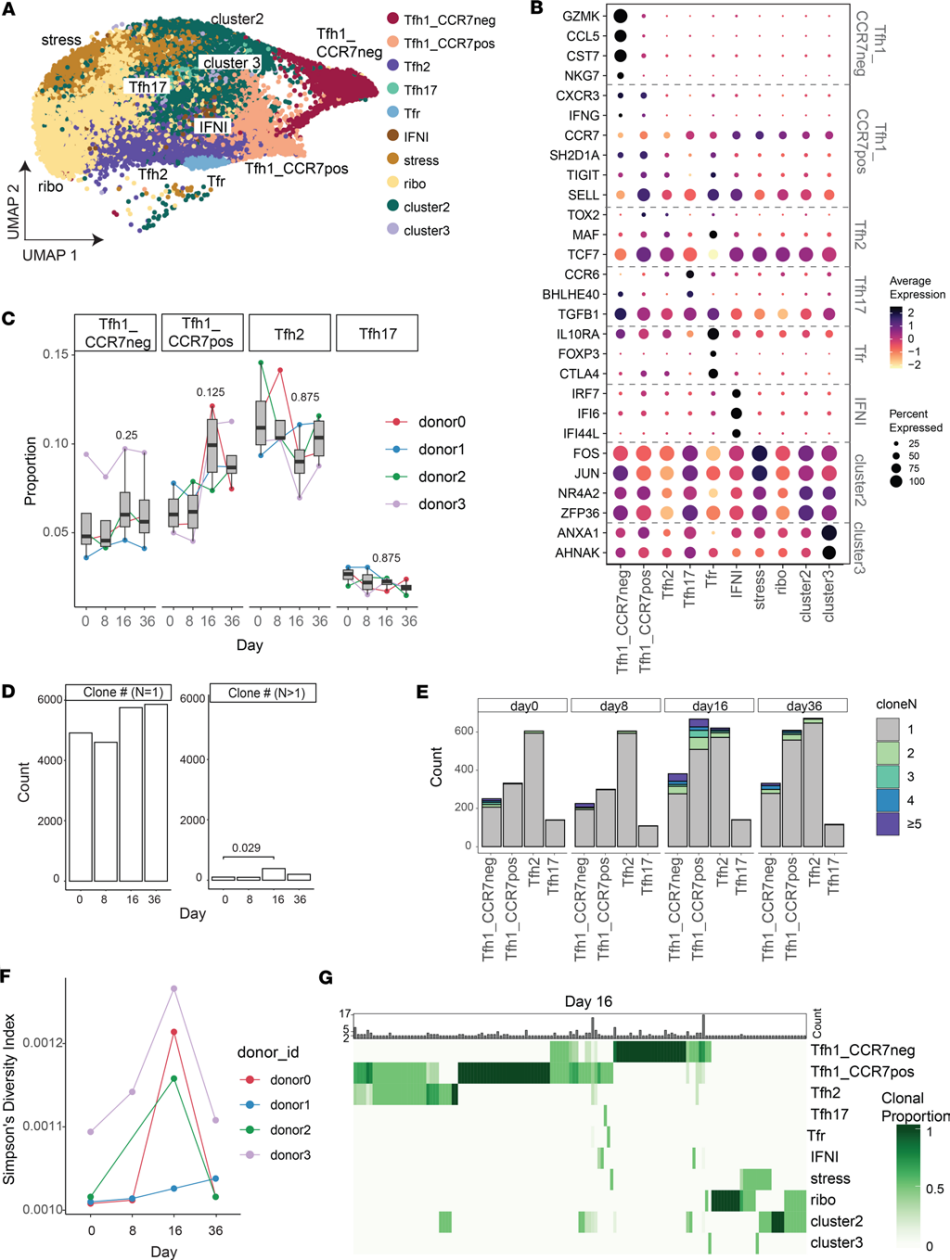
Clonal expansion of diverse pTfh cell subsets during malaria infection. (**A**) pTfh cells (CXCR5^+^PD1^+^ nnCD4 T cells) were isolated from individuals (*n* = 4) during controlled human malaria infection (CHMI) at day 0, 8, 16, and 36. scType label transfer was used to identify Tfh transcriptional subsets using the healthy Tfh cell dataset as a reference. (**B**) Average expression of marker genes used to annotate reference dataset in each subset in the CHMI dataset. (**C**) Relative proportion of each pTfh transcriptional cluster during CHMI. Each individual is shown. Wilcox signed-rank test is indicated for comparison between day 0 and day 16; however, these should be interpreted cautiously due to limited sample size in scRNA-Seq data set. (**D**) Counts of clones with either a clone size = 1 or clone size ≥ 2 across days. Clonal expansion was detected at day 16. (**E**) Clone family size count over time in each subset. Clonal expansion was detected in Tfh1_CCR7^neg^, Tfh1_CCR7^pos^, and Tfh2 clusters at day 16. (**F**) Simpson’s diversity index in each donor across time. (**G**) Clonal overlap at day 16 across clusters. Clonal size is indicated in count in top bar, and each clonal proportion across subsets is indicated. See also Supplemental Figures 6 and 7.

To investigate the roles of pTfh1-CCR7^+^ and pTfh1-CCR7^–^ cells, along with other pTfh cells, during malaria, we applied findings to a CHMI study. pTfh cells (CD45RA^–^CXCR5^+^PD1^+^) were sorted from individuals in CHMI at day 0 (prior to inoculation), day 8 (peak infection), day 15, and day 36 (both after antiparasitic drug treatment) and analyzed using 5′ droplet scRNA-Seq (*n* =4, 100% males, age 22 [21.5–26], median [IQR] years, Supplemental Figure 5, A and B). After QC, 27,552 cells were analyzed, with high-quality cells captured from each donor and time point (day 0 *n* = 6,741, day 8 *n* = 6,125, day 16 *n* = 8,103, day 36 *n* = 7,583, Supplemental Figure 5C). Data were integrated (Supplemental Figure 5D), and pTfh clusters were identified by label transfer with scType (an automated cell type identification tool) (39), using the top 20 upregulated and downregulated marker genes for each cell cluster from the healthy map data (Supplemental Table 5 and Supplemental Figure 5, E–H). scType scores were used to collapse clusters into subsets (Figure 4A and Supplemental Figure 5H). This approach identified Tfh1-CCR7^–^, Th1-CCR7^+^, Tfh2, Tfh17, IFNI, stress, ribosomal, cluster 2, and cluster 3 subsets. Tfh1.17 and Tfr cells were not included in the reference data because of overlapping signatures with Tfh1/Tfh17 and Tfh2 subsets. Following label transfer, Tfh1.17 could not be identified manually; however, Tfr cells were identified by inspection of *FOXP3*, *CTLA4*, and *IL10RA* (Figure 4B). Gene expression of annotated clusters was consistent with reference data (Figure 4B, Supplemental Figure 5G, and Supplemental Table 6).

Within Tfh cell clusters there was a trend of expansion of the Tfh1-CCR7^+^ cluster in all individuals at day 16 and a smaller expansion of Tfh1-CCR7^–^ cells (Figure 4C, Wilcoxon signed-rank test, *P* = 0.125 for Tfh1-CCR7^+^, *P* = 0.25 for Tfh1-CCR7^–^; statistical comparison should be interpreted cautiously due to limited sample size *n* = 4). Tfh2 cells expanded in 3 of 4 individuals at day 8, consistent with the earlier activation of this subset reported previously (13). Other subsets were not clearly impacted by infection (Supplemental Figure 5I). Within each subset, TCRA/B sequences were analyzed. TCR sequences with complete TRA/TRB genes were captured for 21,939 (77.97%) cells, which was comparable across days/cell types (Supplemental Figure 6A). During CHMI there was evidence of clonal expansion at day 16, with a significant increase in clonal family size greater than 1 (Figure 4, D and E), and increased Simpson Index, indicating increased clonal expansion (Figure 4F). Expansion was highest in both the Tfh1-CCR7^–^ and Tfh1-CCR7^+^ clusters, consistent with the increased frequency of these cells and indicative of clonal proliferation (Figure 4E and Supplemental Figure 6B). Clonal expansion was also detected within the Tfh2 cluster at day 16 (Figure 4E). Minimal clonal sharing was detected between donors, with only a single clone shared between donor 0 and donor 1 on day 36, which may indicate sequence convergence detected due to analysis of clones with amino acid rather than nucleotide sequence (40, 41) (Supplemental Figure 6C). All other clonal sharing occurred within an individual, the majority at a single time point (Supplemental Figure 6D). For donor 3, clones were also shared across time points, including day 0 prior to malaria, with large clonal families maintaining a single fate across time in the Tfh1-CCR7^–^ cluster, possibly indicating expansion during a prior infection (Supplemental Figure 6D). At day 16, when malaria-associated expansion was evident, clonal sharing across Tfh clusters was analyzed. While there was a minimal sharing between Tfh1-CCR7^–^ and Tfh2 clusters, the Tfh1-CCR7^+^ cluster shared clones with both the Tfh1-CCR7^–^ and Tfh2 clusters, consistent with the overlapping transcriptional profile of the Tfh1-CCR7^+^ cluster with both Tfh1-CCR7^–^ and Tfh2 cells (Figure 4G). This pattern of clonal sharing may suggest that Tfh1-CCR7^+^ cells can develop into either Tfh2 or Tfh1-CCR7^–^ cell phenotypes, but formal tracking of Tfh subset development at the clonal level is required to confirm this hypothesis.

### Unique transcriptional activation of Tfh1 clusters during human malaria infection.

**Figure 5:**
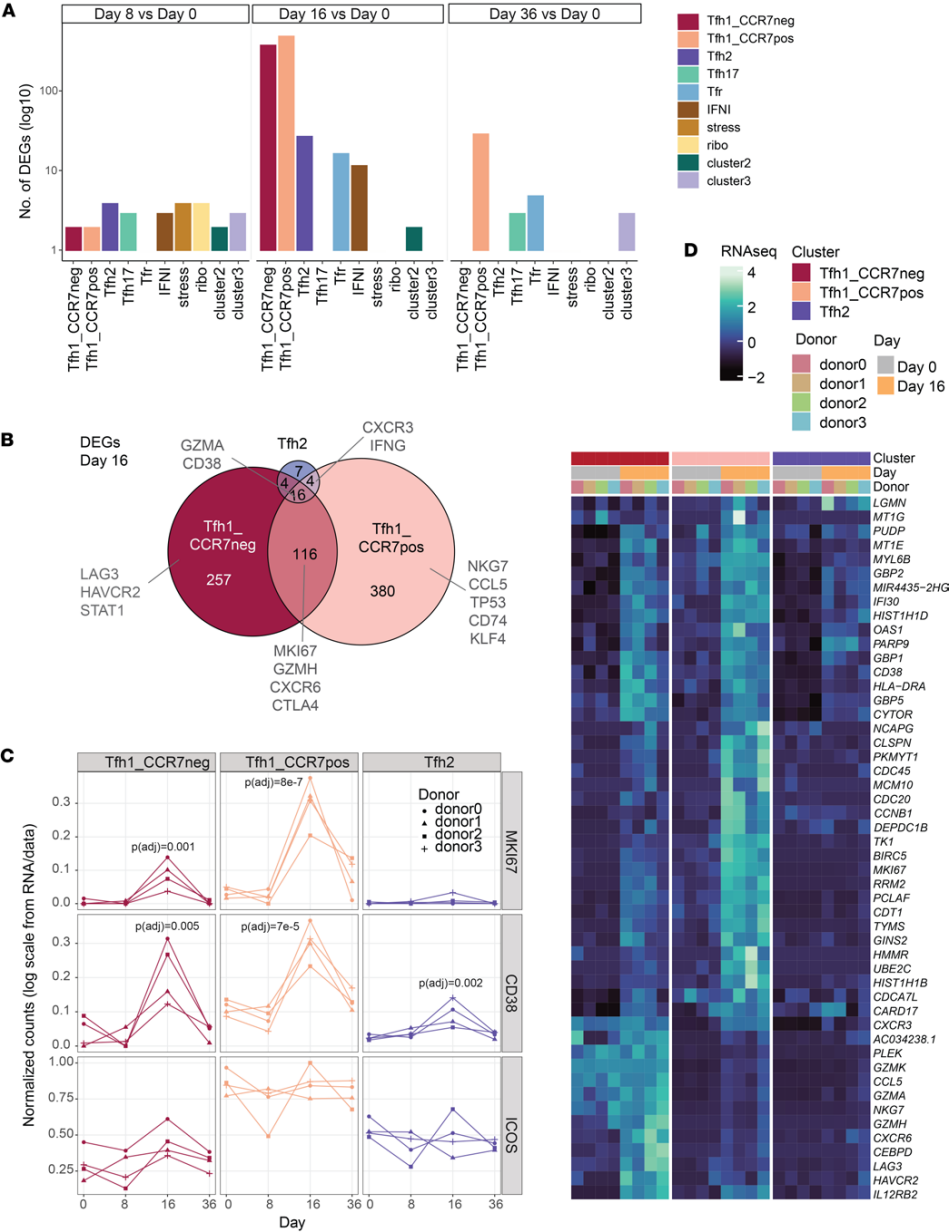
Transcriptional activation of Tfh cell subsets during CHMI. (**A**) DEGs for each cluster were calculated at day 8, 16, and 36 compared with day 0. The number of DEGs for each subset and time point. (**B**) Venn diagram indicating the shared and unique DEGs at day 16 for Tfh1-CCR7^neg^, Tfh1-CCR7^pos^, and Tfh2 subsets. (**C**) Expression of activation genes MKI67, CD38, and ICOS in each individual in each Tfh subset at day 0, 8, 16, and 36 of CHMI. *P* is adjusted from pseudobulk analysis compared with day 0. (**D**) Average expression of top 50 unique upregulated genes for Tfh1-CCR7^neg^, Tfh1_CCR7^pos^, and Tfh2 (curated from top 20 upregulated DEGs in Tfh1-CCR7^neg^, Tfh1-CCR7^pos^, and Tfh2) cells across all 3 subsets at day 0 and at 16 subsets. See also Supplemental Figure 8.

To investigate transcriptional pathways activated in each Tfh transcriptional cluster during malaria, DEGs between day 0 and subsequent time points were identified with pseudo-bulk analysis, grouped by individual (Supplemental Table 7). The largest transcriptional changes were detected at day 16 within the Tfh1-CCR7^+^ and Tfh1-CCR7^–^ clusters (Figure 5A). DEGs at day 16 were also detected in the Tfh2 cluster, consistent with clonal expansion of these cells and previously reported findings (13) (Figure 5A). The number of DEGs was contracted by day 36, with DEGs detected at this time point restricted to the Tfh1-CCR7^+^ cluster, suggesting this cluster had a sustained response to infection. The majority of DEGs at day 16 were cluster specific (Figure 5B). Shared across all 3 clusters was the activation marker *CD38*, and both the Tfh1-CCR7^–^ and the Tfh1-CCR7^+^ clusters also both upregulated *MKI67* (encoding Ki67), consistent with proliferation and clonal expansion of these cells during malaria. However, overall expression of *CD38* and *MKI67* genes was highest in the Tfh1-CCR7^+^ cluster. While *CD38* was upregulated during CHMI, *ICOS* was not upregulated in any subset (Figure 5C and Supplemental Figure 7A). Analysis of the top 20 upregulated genes from the Tfh1-CCR7^+^, Tfh1-CCR7^–^, and Tfh2 clusters highlighted the uniquely upregulated gene signatures identified from Tfh1-CCR7^+^ and Tfh1-CCR7^–^ cells (Figure 5D and Supplemental Figure 7D). Additionally, while upregulated genes also included genes associated with increased inflammation and cytotoxicity, including *NKG7*, *CCL5*, *GZMH*, *CXCR3*, and *IFNG*, the level of these genes was higher in the Tfh1-CCR7^–^ cluster even after infection (Figure 5D and Supplemental Figure 7, B and D). Further, the transcriptional profile of the Tfh2 cluster was also maintained and similar to day 0, despite upregulation of Tfh1-associated genes (Supplemental Figure 7C). Taken together, scRNA-Seq analysis of Tfh cells during malaria identifies the activation, proliferation, and clonal expansion of Tfh1-CCR7^+^, Tfh1-CCR7^–^, and Tfh2 cells with unique transcriptional activation profiles.

### Frequency and proliferation of malaria-specific Tfh-CCR7^+^ cells are associated with the magnitude and function of antibody induction in CHMI.

**Figure 6:**
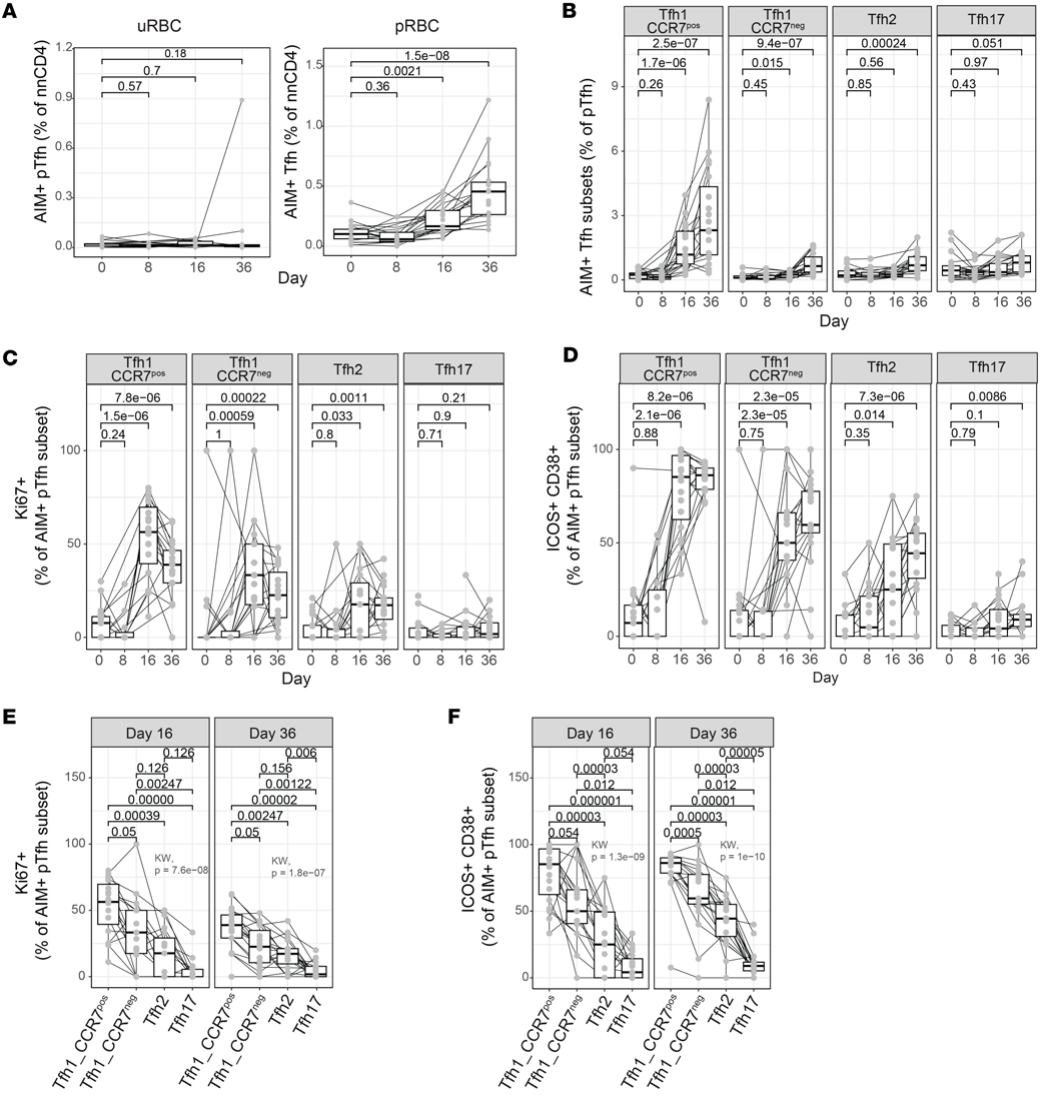
Activation of malaria-specific Tfh cells during malaria. (**A**) Activation induced marker (AIM) assays were used to detected malaria-specific pTfh cells in individuals following CHMI (*n* = 15–20). AIM^+^ cells were identified as CD69^+^OX40^+^ pTfh cells after stimulation with uninfected or parasite-infected RBCs (uRBC and pRBC, respectively) within all non-naive CD4 T cells. Malaria-specific Tfh cells increased at day 16 and day 36 after infection. (**B**) Malaria-specific (AIM^+^) subset-specific pTfh cells as a proportion of total pTfh cells. (**C** and **D**) Ki67^+^ (**C**) and ICOS^+^CD38^+^ (**D**) cells as a percentage of each AIM^+^ malaria-specific pTfh subset during CHMI comparing changes with infection. (**E** and **F**) Ki67^+^ (**E**) and ICOS^+^CD38^+^ (**F**) cells as a percentage of each AIM^+^ malaria-specific pTfh subset at day 16, comparing expression levels between subsets. (**A**–**D**) *P* is the Wilcoxon rank-sum test for each infection time point against baseline. (**E** and **F**) *P* is the Wilcoxon rank-sum test after adjustment with multiple-testing correction using Holm’s method, while the Kruskal-Wallis test is used for the global comparison. See also Supplemental Figure 9.

To confirm transcriptional findings at the phenotypic and antigen-specific level and assess associations of different Tfh subsets with antibody induction, we applied our flow cytometry panel to identify Tfh subsets, including Tfh1-CCR7^+^ and Tfh1-CCR7^–^ cells, to assess the activation of Tfh cells during CHMI in a subset of individuals from a previously published cohort (*n* = 19, sex 83.3%% males, aged 24 years [20.2–28.2, median [IQR] years) (13). Here we expanded on the published analysis by focusing on malaria-specific Tfh cells, which were identified by activated induced marker (AIM) assays following stimulation with parasite-infected RBCs (pRBCs) (17) (Supplemental Figure 9A). Analysis included all non-naive CD4^+^ T cells, including TEMRA cells, which were not included in the scRNA-Seq analysis; however, these cells were a small proportion of the total Tfh response and did not expand following malaria (Supplemental Figure 9B). There were no significant changes in the proportion of Tfh subsets following CHMI; however, there was a trend toward slightly increased Tfh2 at day 8 and slightly increased Tfh1-CCR7^+^ cells at day 14, consistent with prior reports in a larger study of this cohort (13) (Supplemental Figure 9C). AIM^+^ (CD69^+^OX40^+^) Tfh cells were expanded at day 16 and 36 during CHMI. The frequencies of cells detected in control-stimulated cultures (uninfected RBCs, uRBCs) were unchanged (Figure 6A). The largest response of malaria-specific (AIM^+^ in pRBC stimulated) Tfh cells occurred in Tfh1-CCR7^+^ cells; however, increased frequencies of Tfh1-CCR7^–^ and Tfh2 cells were also detected, consistent with the proliferation and expansion of these cells detected transcriptionally (Figure 6B). The frequencies of AIM^+^ Tfh cells were the highest at day 36, possibly suggesting continued emergence of memory cells from secondary lymphoid tissues following activation within the GC (Figure 6A). Indeed, AIM^+^ Tfh2 cells were not detected until day 36, and only a very small population of AIM^+^ Tfh1-CCR7^–^ cells was detected at day 16 (Figure 6B). Increased frequencies of AIM^+^ Tfh17 cells were also detected at day 36 (*P* = 0.051); however, given that Tfh17 cells did not expand clonally, or have any detectable transcriptional changes during infection, these cells are likely to be TCR-independent cells, which are activated via cytokines within AIM assays (42). Within malaria-specific Tfh1-CCR7^+^, Tfh1-CCR7^–^, and Tfh2 cells, there were significant increases in expression of activation markers ICOS and CD38 and proliferation marker Ki67 at day 16 and 36 (Figure 6, C and D). However, the expression of these markers was significantly higher on malaria-specific Tfh1-CCR7^+^ cells compared with other subsets, consistent with the greatest expansion and activation of Tfh1-CCR7^+^ cells transcriptionally (Figure 4C and Figure 6, E and F).

**Figure 7:**
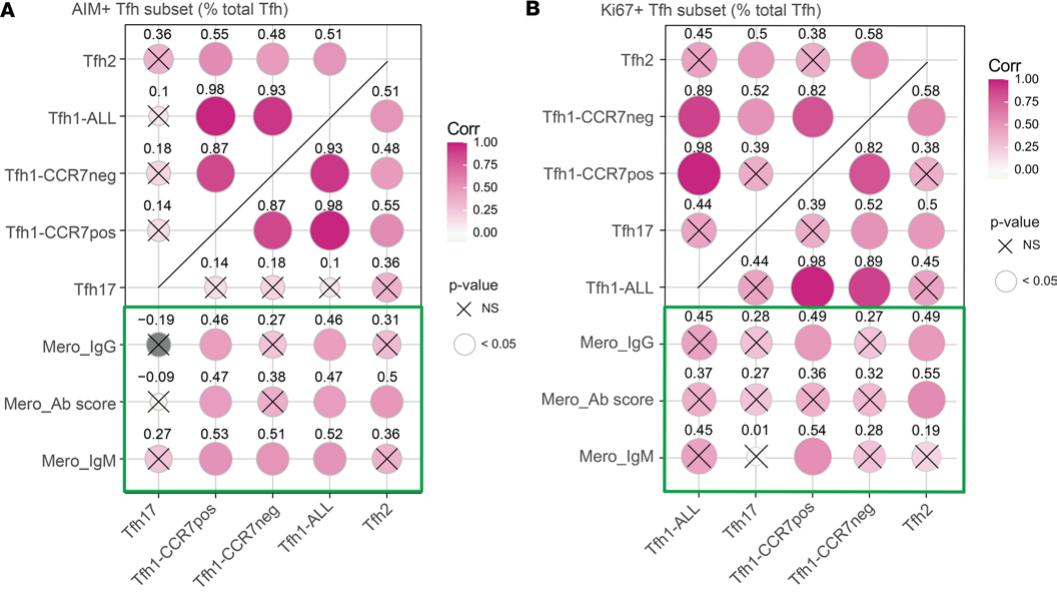
Associations between malaria-specific or proliferating Tfh and antibody induction. Correlation between (**A**) AIM^+^ malaria-specific pTfh cell subsets or (**B**) Ki67 expression of Tfh subsets at day 36 with total IgG and IgM levels against merozoite and overall antibody score against merozoite, which captures the total antibody magnitude, breadth, and functionality specific to merozoite. Tfh1-ALL is a combination of Tfh1-CCR7^neg^ and Tfh1-CCR7^pos^ cells. Spearman’s rho and *P* value are indicated. See also Supplemental Figures 9 and 10.

To investigate the link between each malaria-specific Tfh cell subset and antibody development, we correlated malaria-specific Tfh cells with antibodies produced after infection in response to intact merozoites, which were quantified previously (13). Antibody development was quantified with an antibody score that captured the magnitude, breadth, and functionality of response, by measuring merozoite-specific IgM, IgG, and IgG subclasses; C1q fixation; FcR cross-linking; and opsonic phagocytosis (13). We analyzed correlations between these antibody responses and AIM^+^ Tfh cells at day 36, when responses in the periphery were maximal, which we hypothesize is the best measure of functional responses earlier in infection within lymphoid tissues (Figure 6B). The frequencies of malaria-specific Tfh1-CCR7^+^, Tfh1-CCR7^–^, and Tfh2 cells at day 36 correlated with each other but not with Tfh17 cells (Figure 7A). Despite this, Tfh1-CCR7^+^ cells were correlated the most strongly and consistently with antibody induction compared with other subsets and were positively correlated with total antibody score, and IgM and IgG against the merozoite (Figure 7A), along with IgG1 and IgG2 (Supplemental Figure 8D). In contrast, Tfh1-CCR7^–^ cells only correlated with IgM and pTfh2 cells only with total antibody score (Figure 7A). Similarly, while Ki67 expression among subsets was correlated, only Ki67^+^ Tfh1-CCR7^+^ and Ki67^+^ Tfh2 cells were correlated with antibody score, IgG and IgM, and antibody functions (Figure 7B and Supplemental Figure 8, D–G). We also analyzed associations between antibodies and the total Tfh1 compartment, combining both Tfh1-CCR7^+^ and Tfh1-CCR7^–^ cells. While AIM^+^ total Tfh1 cells associated with the antibody response, Ki67^+^ total Tfh1 cells did not, consistent with a potential role of CCR7 expression in Tfh1 cell function (Figure 7A). No significant correlations were found between Tfh subsets at day 16 and antibodies at EOS, consistent with the continued emergence of malaria-specific Tfh cells between day 16 and 36 (all Spearman’s correlations *P* > 0.05). Together, data highlight that Tfh1-CCR7^+^ and Tfh1-CCR7^–^ cells have distinct functional potential. While both are activated in malaria, Tfh1-CCR7^+^ cells, together with Tfh2 cells, are more strongly associated with antibody development.

## Discussion

Here we provide data that advance understanding of Tfh cell subsets, highlighting their functional diversity across the course of a human malaria infection. Using unbiased analysis to integrate Tfh in the resting state, we identify 2 distinct Tfh1 subsets, which can be distinguished based on CCR7 expression phenotypically. Tfh1-CCR7^+^ cells do not express cytotoxic markers and produce less inflammatory cytokines but similar IL-21 levels compared with Tfh1-CCR7^–^ cells. Expanding our findings to the context of human infection, we show during malaria that while Tfh1-CCR7^+^, Tfh1-CCR7^–^, and Tfh2 cell subsets expand and are activated, transcriptional activation pathways are unique between the subsets. We show, both transcriptionally and at the malaria-specific and phenotypic level, the magnitude of activation is greatest in Tfh1-CCR7^+^ cells, which also expand clonally. Additionally, the frequency and proliferation of Tfh1-CCR7^+^ cells were most consistently associated with antibody induction compared with other subsets. Taken together, these findings provide knowledge on Tfh cell activation and functions during human infection. The specific role of 2 distinct Tfh subsets, Tfh1-CCR7^+^ and Tfh2 cells, in antibody induction during malaria, informs approaches to target Tfh cells to improve antibody development and protection.

Understanding the mechanisms driving the development of distinct Tfh cell subsets has important translational implications for vaccine design and therapeutic interventions. Development of Tfh cell subsets is suggested to be mediated by cytokine skewing driven by distinct inflammatory milieu occurring during different immune responses to infection or vaccination (5). A recent investigation of Tfh cell development in mice immunized with type 1– or type 2–adjuvanted conditions supported this concept, inducing Tfh1 or Tfh2 cell–dominated responses, respectively (33). However, a comparative investigation of Tfh cell development and function in different infections has highlighted that specific cytokine signaling pathways within Tfh cells is more important in driving subset fate than pathogen class (8). For example, within that study, Tfh cell fates induced by LCMV and influenza, both of which drive type 1 immune responses, were distinct and drove differing antibody responses (8). Here, our data highlight that even within a single infection, induced Tfh cells are diverse. During malaria both Tfh1-CCR7^+^ and Tfh1-CCR7^–^ subsets, along with Tfh2, are expanded, transcriptionally activated, and malaria specific. Tfh1-CCR7^+^ cells shared clonal overlap with both Tfh1-CCR7^–^ cells and Tfh2 subsets, but these latter subsets were largely clonally distinct from each other. These findings may indicate that Tfh1-CCR7^+^ cells can develop into either Tfh1-CCR7^–^ or Tfh2 cell subsets or arise from these populations or a shared precursor population. Alternatively, clonal sharing may be underpinned by a temporary nature of Tfh cell phenotypes identified, or plasticity between subsets. CD4^+^ T cell plasticity is well recognized; however, the mechanisms driving plasticity, including the roles of stem-like CD4^+^ T cells, which share many characteristics with Tfh cells, is a topic of ongoing debate (43). While the shared clonal relationships of malaria-specific Tfh cells across subsets are supported by other studies (44), whether these relationships indicate cells along a single developmental pathway, or divergence from a parent population, and how cytokine signaling may influence subsets development are unknown.

The importance of understanding Tfh cell subset development is highlighted by the distinct functional consequences on antibody development linked to Tfh cell subset function. Human studies linking Tfh cell subsets to antibody development have largely been limited to identifying Tfh cell subsets based on CXCR3 and CCR6 expression (9, 10, 45, 46), including in malaria (13, 17, 18, 20, 44). Alternatively, studies that include a more comprehensive analysis of Tfh cells have not related Tfh cell diversity to antibody development (32, 47). Here, our scRNA-Seq analysis shows that while CXCR3 and CCR6 are informative, diversity is masked by this gating strategy. Of functional relevance, within Tfh1 cells, we found that Tfh1-CCR7^–^ cells contain the large majority of Tfh cells expressing cytotoxic markers, including *NKG7*, granzyme K, and granzyme B. In contrast, these markers were absent from Tfh1-CCR7^+^ cells, which produced less inflammatory cytokines but comparable levels of IL-21, resulting in a distinct cytokine milieu. Tfh cells with expression of cytotoxic markers have been identified previously, and GC Tfh cells that produce granzyme B in response to group A *Streptococcus* kill B cells, potentially hampering antibody development (48). More recently granzyme K^+^ CMV-specific Tfh cells were reported in humans. However, the function of these cells in the context of antibody development is unknown (33). During malaria, we show that while malaria-specific Tfh1-CCR7^+^, Tfh1-CCR7^–^, and Tfh2 cell subsets are induced, only Tfh1-CCR7^+^ and Tfh2 cell subsets were clearly associated with antibody development. We have previously reported in the same cohort that global Tfh2 cell activation early in infection (not considering antigen specificity) was associated with antibody development (13), a finding supported by others (14–16). However, a negative role of Tfh1 in antibody development was difficult to rationalize in the context of what is known from in vitro studies and animal models (21, 49, 50) of the link between Tfh1 cells, T-bet–expressing B cells, and induction of IgG3 antibodies, which are consistently associated with protection from malaria in cohort studies (22). Further, our data from high-transmission areas in Uganda showed that children who had the highest levels of functional antibodies had increased proliferating Tfh2 and Tfh1 cells (20). Data presented here contribute to resolving these contradictions. Further studies are required to investigate whether Tfh1-CCR7^–^ cells with cytotoxic functions have negative roles in antibody induction and how Tfh1-CCR7^+^ and Tfh2 cells function to induce protective antibodies in malaria. Nevertheless, data highlight that the standard analysis of Tfh subsets based on CXCR3 and CCR6 expression overlooks important functional differences, particularly within Tfh1 cells. We propose that future studies should consider analysis of Tfh cells based on CXCR5, and PD-1, together with a minimal inclusion of CXCR3, CCR6, CCR7, CD45RA, and activation markers (a selection of CD38/ICOS/Ki67), to fully capture the diversity of responses and associations with antibody induction.

Limitations of our study include the analysis of Tfh cells isolated from blood of volunteers, not within secondary lymphoid tissues. While fine needle aspirate studies of lymph nodes during human infection and vaccination have been used to investigate GC responses in influenza and COVID-19 (51–58), to date these approaches have not been applied to malaria. It remains to be determined how the profiles of Tfh cells in the blood represent GC responses and whether timing of Tfh analysis used here is relevant for function within the GC during malaria. Additionally, while Tfh cells share overlapping signatures with GC Tfh cells and are thought to provide a snapshot of the GC response (6), activated Tfh may not fully represent all GC Tfh phenotypes and functions (59). Future studies will focus on developing approaches for fine needle aspirate studies of draining lymph nodes during human malaria as applied to COVID-19 and influenza (52, 54, 56, 59, 60) and investigating the immune response within the spleen in malaria infected individuals (61, 62). Nevertheless, such approaches are not feasible for large human cohorts; thus, data here on Tfh cells provide important insights that can be applied to other human cohort studies. Additionally, while phenotypic analysis of Tfh1 CCR7^+/–^ subsets suggested functional differences and we provide in vivo evidence of differential associations with antibody development consistent with differences in B cell helper capacity, future studies are required to test in vitro if Tfh1 CCR7^+/–^ subsets can provide different levels of B cell help in mixed lymphocyte reactions or have cytotoxic capacity.

In conclusion, this study provides important knowledge on functional diversity within Tfh cells during human infection. We identify specific Tfh cell subsets that associate with antibody induction in malaria, thus informing the development of approaches that can target these cells to improve antibody development induced during vaccination. We provide an scRNA-Seq data set that can be applied to other diseases and an optimized approach to identify distinct Tfh1 subsets based on CCR7 expression in large human clinical cohorts. The identification of further Tfh subsets and their potential functional importance is highly relevant to developing novel therapeutics or new vaccine strategies that can induce potent and long-lasting immunity against not only malaria but also other important pathogens.

## Methods

### Sex as a biological variable

Both males and females were included in this study. For some parent CHMI studies, females of childbearing age were excluded, and as such the sex distribution is not evenly distributed.

### Study cohorts

CHMI studies were performed as previously described (63). In brief, healthy malaria-naive individuals underwent CHMI with 2,800 viable 3D7 strain *P*. *falciparum* pRBCs, and peripheral parasitemia was measured by quantitative PCR (64). Participants were treated with antimalarial drugs at day 8 of infection, when parasitemia reaches approximately 20,000 parasites/mL. Blood (5 studies, 6 independent cohorts) was collected prior to infection (day 0), at peak infection (day 8), and 14 or 15 and 27–36 days (end of study, EOS) after inoculation (in analyses these time points are grouped as 0, 8, 14/15, and EOS). PBMCs were isolated by Ficoll-Paque (Sigma) density gradient centrifugation; isolated PBMCs were cryopreserved in 10% DMSO/FBS. Participants were healthy malaria-naive adults with no prior exposure to malaria or residence in malaria-endemic regions. Clinical trials were registered at ClinicalTrials.gov NCT02867059 (65), NCT02783833 (66), NCT02431637 (67), and NCT02431650 (67). For trials NCT02867059, NCT02783833, and NCT02431637 all participants received the same study drug (no randomization). For NCT02431650, participants were randomized 1:1 to receive study drug or control. Samples used here were opportunistically collected from volunteers who consented to immunological studies within the parent clinical trial. As such, no sample size estimation was performed for this immunology study.

PBMCs from healthy noninfected controls were collected by the same processes for analysis of responses following PMA/Io stimulation and transcriptional map of pTfh cells in healthy donors.

### Parasite culture

Packed RBCs, acquired from Australian Red Cross, Melbourne, Australia, were infected with the *P*. *falciparum* 3D7 parasite strain (68) and cultured at 5% hematocrit in RPMI 1640 (Gibco) supplemented with AlbuMAX II (Thermo Fisher Scientific) (0.25%) and heat-inactivated human sera (5%), at 37°C in 1% O_2_, 5% CO_2_, 94% N_2_ gas mixture. Culture medium was replaced daily, and parasites were monitored by Giemsa-stained blood smears. pRBCs were grown to 15% parasitemia and purified from uRBCs via magnet separation to enrich mature trophozoite stage pRBCs. Purified pRBCs (>95% purity) were stored at 80°C in glycerolyte cryopreservant. For use in stimulations, glycerolyte-preserved parasites or control uRBCs were thawed by dropwise addition of malaria thawing solution (MTS: 0.6% NaCl in H_2_O) and incubated at room temperature (RT) for 10 minutes. Parasites were washed in MTS/PBS 3 times with decreasing solution ratios (1:0, 1:1, 0:1). Following thaw, parasites were intact late stage trophozoites.

### Ex vivo cell phenotyping with flow cytometry and AIM assays

PBMCs were thawed in RPMI 1640 containing 10% FCS and 0.02% Benzonase Nuclease (Merck) and rested for 2 hours at 37°C, 5% CO_2_, and then stimulated for 18 hours in 96-well plates in 10% FCS/RPMI at 1 × 10^6^ cells per well with pRBCs or uRBCs at 1:1 ratio. pRBC stimulated cells were used to assess malaria-specific responses, and uRBC control-stimulated cells were used to phenotype all Tfh cells. To understand Tfh cells in healthy donors, we analyzed PBMCs in individuals in CHMI studies prior to inoculation, and to understand responses to malaria, we analyzed PBMCs at postinfection time points. PBMCs were stained with antibodies against CD49b and LAG3 (Invitrogen) along with Fc block (BD Biosciences) for 45 minutes at 37°C, 5% CO_2_. Following 2 washes with PBS, cells were stained with LIVE/DEAD Fixable Blue Dead Cell Stain (Thermo Fisher Scientific) for 15 minutes at RT, washed twice with 2% FCS/PBS, and then surface-stained for 15 minutes at RT with fluorescent-tagged antibodies from BioLegend, and BD Biosciences (Supplemental Table 3). After 2 washes with 2% FCS/PBS cells were fixed and permeabilized with eBioscience Foxp3/Transcription Factor Staining Buffer Set (Thermo Fisher Scientific; catalog 00-5523-00) for 20 minutes on ice. After 2 washes with 1× perm buffer, intracellular staining was performed for 30 minutes on ice (Supplemental Table 3). Cells were resuspended in 2% FCS/PBS, and events were collected on the Cytek Aurora 5 laser cytometer.

### PMA stimulation and intracellular cytokine staining

Approximately 1 × 10^6^ PBMCs were used per condition. For cytokine induction following PMA/Io stimulation, PBMC samples were rested overnight in 10% FCS/RPMI and then stimulated with PMA (25 ng/ mL; Sigma) and Io (1 μg/mL; Sigma) for 6 hours at 37°C, 5% CO_2_. Brefeldin A (10 μg/mL; BD Biosciences) and monensin (10 μg/mL; BD Biosciences) were added after 2 hours. Antibodies against CCR7 (BD Biosciences) CXCR3 (BD Biosciences) CXCR5 (BioLegend), and CCR6 (BD Biosciences) were added alongside Fc block during stimulation, prior to addition of Brefeldin A and monensin. After stimulation, cells were washed with PBS and stained at RT for 15 minutes with LIVE/DEAD Fixable Blue Dead Cell Stain, washed twice with 2% FCS/PBS, and then surface-stained with the antibodies listed in Supplemental Table 4 for 15 minutes. Following 2 washes with 2% FCS/PBS, cells underwent fixation/permeabilization with eBioscience Fixation/Permeabilization solution for 20 minutes on ice and washed in permeabilization buffer. Intracellular staining with the antibodies listed in Supplemental Table 4 was performed for 30 minutes on ice and then cells fixed with BD stabilizing fixative. Samples were acquired on Aurora 5.

### Flow cytometry data analysis

Flow cytometry was analyzed using FlowJo v10. Unsupervised data analysis was performed with spectre (version 1.2.0) (69).

### scRNA-Seq and analysis

#### Cell isolation: healthy reference dataset.

PBMCs from 4 healthy donors were thawed in RPMI 1640 containing 10% FCS and 0.02% Benzonase. Cells were rested for 2 hours in 10% FCS/PBS at 37C, 5% CO_2_. Untouched CD4^+^ T cells were enriched from PBMCs using the CD4^+^ T cell isolation kit (Miltenyi Biotec). Enriched CD4^+^ T cells were incubated with Fc block for 15 minutes and surface-stained with antibodies listed in Supplemental Table 8 for 15 minutes at RT. Cells were washed with 0.5% BSA/PBS and stained for viability with Sytox Blue (Invitrogen, catalog S34857) and sorted on BD FACSAriaIII Cell Sorter into 0.5% BSA/PBS. Four populations of cells were sorted, PD1^–^ Tfh (non-naive [nn] CD4/CXCR5^+^PD1^–^), pTfh1 (nnCD4/CXCR5^+^PD1^+^CXCR3^+^CCR6^–^), pTfh2 (nnCD4/CXCR5^+^PD1^+^CXCR3^–^CCR6^–^), and pTfh17 (nnCD4/CXCR5^+^PD1^+^CXCR3^–^CCR6^+^). After sorting, cells of a single phenotype were pooled and run on a single 10x Genomics Chromium lane.

#### Cell isolation: CHMI dataset.

PBMCs collected at day 0, 8, 16, and 36 from 4 participants in CHMI study NCT02867059 (65) were thawed in RPMI 1640 (Gibco) containing 10% FCS and 0.02% Benzonase. Cells were washed with 0.5% BSA/PBS and then incubated with Fc block and antibodies against pan-γδ and CXCR5 for 15 minutes at RT. Following that, the other antibodies listed in Stain 2 in Supplemental Table 9 were added, and cells were incubated for another 15 minutes at RT. Cells were washed and stained for viability with Sytox Blue (Invitrogen, catalog S34857) and sorted on BD FACSAriaIII Cell Sorter into 0.5% BSA/PBS. Live Tfh cells were sorted as nnCD4/CXCR5^+^PD1^+^ cells. After sorting, cells from a single time point were pooled and run on a single 10x Genomics Chromium lane.

#### 10x Genomics Chromium GEX library preparation and sequencing.

Sorted cells were loaded into each lane of Chromium Next GEM Single Cell 5’ Reagent Kit v2 and Gel Bead-in-Emulsion (GEMs) generated in the 10x Chromium Controller [Chromium Next GEM Single Cell 5’ Kit v2, PN-1000263, Chromium Single cell V(D)J Amplification Kits: Human TCR, PN-1000252, Chromium Next GEM Chip K Single Cell Kit, PN-1000286, Dual Index Kit TT Set A, PN-1000215] 5’ Gene Expression.

Libraries and VDJ libraries (TCR) were generated according to the manufacturer’s instructions. Generated libraries were sequenced in a NextSeq 550 System using High Output Kit (150 Cycles) version 1 according to the manufacturer’s protocol using paired-end sequencing (150 bp read 1 and 150 bp read 2) with the following parameters: read 1: 26 cycles, i7 index: 10 cycles, i5 index: 10 cycles and read 2: 90 cycles.

#### Demultiplexing and analysis.

Each scRNA-Seq data set was demultiplexed, aligned, and quantified using Cell Ranger version 3.1.0 software (10x Genomics) against the human reference genome (GRCh38-3.0.0) with default parameters. Cell genotype was obtained using cellsnp-lite (70) with the list of informative SNPs obtained from 1000 Genomes Project (71). Then vireo was used to predict the original donor utilizing the cell’s haplotype ratios, and only predicted singlets were used for the analysis (72). Cell Ranger count matrices for each sample were loaded, merged, and analyzed using Seurat package v4.3.0 and v5.1.0 (73) and visualized with scCustomize v3.0.1 (74). Cells were filtered to remove those expressing fewer than 200 genes and more than 4,000 genes and those with more than 10% mitochondrial content. Only genes expressed in 3 or more cells were considered. TRA/TRB gene expression was removed from the gene expression dataset prior to further downstream processing.

For healthy reference map, the dataset was split by donor, and then each individual donor dataset was normalized with SCTransform where highly variable genes were also selected. Before integration, the most variable genes shared among the donor datasets were identified using FindIntegrationAnchors and were used to integrate the datasets using the IntegrateData function. Principal component analysis (PCA) dimensionality reduction was performed using RunPCA, and the top 20 PCs were used to compute UMAP visualization using RunUMAP.

For CHMI data, the dataset was split per donor and day; then each individual donor_day dataset was normalized with SCTransform where highly variable genes were also selected (75). Before integration, the most variable genes shared among the datasets were identified using FindIntegrationAnchors and used to integrate the datasets using the IntegrateData function. PCA dimensionality reduction was performed using RunPCA, and the top 20 PCs were used to compute UMAP visualization using RunUMAP.

#### Cell cluster annotation: healthy reference map.

We applied unsupervised clustering using the Louvain algorithm as our modularity optimization technique and set the granularity of clusters at a resolution of 0.3. A cluster with high mitochondrial content and low read counts was removed. RNA-level counts were log-normalized using NormalizeData (scale factor = 10,000 by default) and were used to run FindMarkers for identifying cluster marker genes as part of cluster annotation. This identified the Tfh1 cluster, along with Tfh2, Tfh17, stress, ribosomal, IFNI, cluster 2, and cluster 3 clusters. We then visualized the expression of Treg-related genes (*FOXP3* and *IL2RA*) to identify a cluster of cells belonging to Tfr, and another subset of Tfh1.17 cluster that expressed *RORC* along with *CXCR3*. To identify DEGs between the 2 Tfh1 clusters, FindMarkers function was applied in Seurat v5, which identifies DEGs with Wilcoxon’s rank sum, identifying genes expressed in at least 0.1% of cells, ≥0.25 log fold-change, followed by Bonferroni’s correction. Automated cell-labeling tool, scType (39), was used to transfer labels from the healthy reference Tfh map onto a published human tonsil Tfh dataset (27) containing non-GC and GC Tfh subsets. Detailed steps of how the label transfer was performed is as per described below in CHMI data section. Cluster markers of each cluster were further analyzed for pathway enrichment using the Ingenuity Pathway Analysis (IPA) suite (v01-22-01, QIAGEN).

#### Cell cluster annotation: CHMI.

Unsupervised clustering was calculated using Louvain algorithm with resolution set at 2.2 to achieve overclustering. Low-quality clusters with high mitochondrial content and low read counts were removed. Label transfer using cell type annotations from the healthy reference map onto the CHMI dataset was performed using scType (39). The top 20 up- and downregulated cluster marker genes from each reference cell type were used to calculate a specificity score to predict the cell type for each cluster in the CHMI dataset. Based on the final top specificity score, similar clusters are then stitched together for the final annotation. As an additional quality check step, a fold-change between the score of the top-predicted cell type versus every other possible cell type was calculated per cluster, and any cluster where the difference between the top-predicted cell type and the next possible cell type was less than 1.1 was flagged as “low-confidence.” For clusters 14, 19, and 22, where the top-predicted cell type was flagged as “low-confidence,” a manual comparison of key cell type marker genes was done to confirm the final cell annotation used (final annotation: cluster 14 as Tfh1_CCR7pos, cluster 19 as cluster 2, cluster 22 as Tfh1_CCR7neg). After the final prediction, we visualized Treg markers (*Il2ra*, *Foxp3*) and manually selected for an area on the UMAP with high expression of these markers as Tfr cluster. For the reference database, we omitted Tfh1.17 and Tfr as input as these 2 clusters had signatures commonly shared with the other clusters. RNA-level counts were log-normalized using NormalizeData (scale factor = 10,000 by default) and were used to run FindMarkers for identifying additional cluster marker genes for each annotated cluster.

#### Pseudobulked differential gene expression analysis and pathway analysis.

The normalized count matrix and the metadata were extracted from the Seurat object. Counts were aggregated per sample (i.e., donor and day) for each cluster. Significant genes were determined using the quasi-likelihood *F* test with an FDR < 0.05 using edgeR (76). Aggregated counts were used for visualizing changes in specific DEGs using ComplexHeatmap.

#### TCRA/B clonal analysis.

Annotated TCR sequences were integrated with the single-cell transcriptomics data using the combineTCR and combineExpression functions from scRepertoire (version 2.0.0). The complementarity-determining region 3 amino acid sequences were used to call and quantify clones. Only cells with single and complete paired TCRA and TCRB chain were included. Clones were defined as cells sharing identical amino acid sequence in both TRA and TRB. The R package circlize (v0.4.16) and clonalOverlap function of scRepertoire were used to generate the Circos and heatmap plots of clonal overlap, respectively. For Simpson’s diversity index calculations, data were downsampled to 1,000 cells to adjust for cell count variations between samples.

### Statistics

Nonparametric testing was performed for all analysis. Continuous data for all cellular responses were compared between groups using Mann-Whitney *U* test or in paired samples with Wilcoxon paired signed-rank test, or correlated with antibody score as a continuous variable with Spearman’s correlations. Statistical comparisons were adjusted for multiple comparisons where indicated. Overall composition of the cytokine responses between Tfh1 subsets was compared using Permutation test with SPICE (77). All analyses were performed in R (version 4.4.2). Graphical outputs were made in ggplot2 (version 3.5.1) and ggpubr (version 0.6.0). No sample size calculation was performed; instead, all available participant data were included. Due to sample size restrictions, no subgroup analysis was performed. Data generation was performed with blinding to participant demographic data. A *P* value of less than or equal to 0.05 was considered significant.

### Study approval

Ethics approval for human studies was obtained from the QIMR Berghofer Human Research Ethics Committee, Brisbane, Queensland, Australia (HREC P1479), and the Alfred Health Ethics Committee, Melbourne, Victoria, Australia (288/23). Written informed consent was obtained from all study participants.

### Data availability

All data generated or analyzed during this study are included in this article and supplement and available in Supporting Data Values file or deposited online as follows. The raw sequencing data and processed Cell Ranger outputs used in this study have been deposited in the National Center for Biotechnology Information database under accession code GSE272939 (https://www.ncbi.nlm.nih.gov/geo/query/acc.cgi?acc=GSE272939) and GSE253661 (https://www.ncbi.nlm.nih.gov/geo/query/acc.cgi?acc=GSE253661).

The processed scRNA-Seq data are available in Zenodo, https://doi.org/10.5281/zenodo.14847353

Code used to analyze scRNA-Seq data is available at https://github.com/MichelleBoyle/CCR7-expression-defines-distinct-pTfh1-subsets-involved-in-malaria-immunity.git (commit ID 125ce2a).

Flow cytometry files are available at Zenodo, https://doi.org/10.5281/zenodo.16947286 and https://doi.org/10.5281/zenodo.16953871

## Author contributions

MSFS, NLD, RM, DWA, JRL, and JAC generated data, supervised by JGB, CRE, AH, and MJB. MSFS, DAO, and ZP analyzed data. JSM provided clinical samples used in this study. AH and MJB conceptualized the study. MSFS and MJB led manuscript writing. All authors contributed to and approved the manuscript.

## Funding support

National Health and Medical Research Council (NHMRC) of Australia (program grant 1132975 to JSM and CRE).

- Senior Research Fellowships (1154265) to CRE.
- Career Development Award 1141278, Project Grant 1125656, and Ideas Grant 1181932 to MJB.
- CSL Centenary Fellowship and Snow Medical Foundation Fellowship 2022/SF167 to MJB.
- Jim and Margaret Beever Fellowship to JAC.
- NHMRC for Independent Research Institutes Infrastructure Support Scheme and the Victorian State Government Operational Infrastructure Support to Burnet Institute.
- Medicines for Malaria Venture to the parent CHMI studies.

## Supplementary Material

Supplemental data

Supplemental tables 1-9

Supporting data values

## Version 1 10/21/2025

In-Press Preview

## Version 2 12/08/2025

Electronic publication
