# Supplementary material for: Tfh2 and a subset of Tfh1 cells associate with antibody-mediated immunity to malaria
Source: JCI Insight. 2025 Oct 21;10(23):e196828. doi: 10.1172/jci.insight.196828 (PMC12890485; doi:10.1172/jci.insight.196828)
Supplement: Supplemental data [file jciinsight-10-196828-s024.pdf]

Supplementary Figures and Tables

Supplementary Tables.

Supplementary Table S1: Cluster markers for pTfh clusters in healthy donors *attached as excel*

Supplementary Table S2: DEGs between Tfh1_cyto and Tfh1_CCR7 clusters *attached as excel*

Supplementary Table S3: Phenotyping panel

Target	Fluorochrome	Clone	Manufacturer	Catalog #	Dilution
LAG-3	BUV661	3DS223H	Invitrogen	376-2239-42	1 in 25
CD49b	FITC	eBioY418	Invitrogen	11-0498-42	1 in 50
CD25	BUV395	2A3	BD Biosciences	564034	1 in 100
CXCR5	BUV615	MU5UBEE	Invitrogen	366-9185-42	1 in 50
CXCR3	BV421	1C6/CXCR3	BD Biosciences	562558	1 in 25
CCR6	BV605	11A9	BD Biosciences	562724	1 in 50
OX40	BV650	ACT35	BD Biosciences	563658	1 in 50
CCR7	BV711	150503	BD Biosciences	566602	1 in 50
CD3	BV786	UCHT1	BD Biosciences	565491	1 in 500
CD4	Spark blue 574	SK3	Biolegend	389714	1 in 100
CD69	PerCP-eFluor 710	FN50	ThermoFisher	46-0699-42	1 in 100
TIM-3	PE-CF594	7D3	BD Biosciences	565560	1 in 50
PD-1	PE-Fire 640	EH12.2H7	Biolegend	329968	1 in 50
TIGIT	PE-Fire 810	A151513G	Biolegend	372745	1 in 100
CD45RA	redFluor 710	HI100	Cytek Biosciences	80-0458-T100	1 in 400
ICOS	APC-H7	DX29	BD Biosciences	567142	1 in 100
CD38	APC-Fire 810	HIT2	Biolegend	303550	1 in 200
Viability	LD-Blue		ThermoFisher	L23105	1 in 5000
**Intracellular:**					
CTLA-4	BUV737	14D3	ThermoFisher	367-1529-42	1 in 50
Ki-67	BUV805	B56	BD Biosciences	569636	1 in 50
Granzyme B	BV510	GB11	BD Biosciences	563388	1 in 800
Granzyme K	RB780	G3H69	BD Biosciences	569225	1 in 50
NKG7	PE	E6S2A	Cell Signalling Technology	84835	1 in 2000
c-Maf	PECy7	sym0F1	ThermoFisher	25-9855-82	1 in 400
FOXP3	RB613	259D/C7	BD Biosciences	571087	1 in 100
SAP	eFluor660	XLP-1D12	ThermoFisher	50-9787-42	1 in 100

Supplementary Table S4: PMA/Io panel

Target	Fluorochrome	Clone	Manufacturer	Catalog #	Dilution
CD8	BUV496	RPA-T8	BD Biosciences	612942	1 in 100
CD45RA	BUV563	HI100	BD Biosciences	612926	1 in 400
CD3	BUV805	SK7	BD Biosciences	612893	1 in 50
CD14/CD19	BV510	M5E2 / SJ25C1	Biolegend	301842/ 363019	1 in 100
CCR4	BV605	L291H4	Biolegend	359418	1 in 100
CCR6	BV650	11A9	BD Biosciences	563922	1 in 50
CXCR5	BV711	J252D4	Biolegend	356934	1 in 50
CD4	BV785	OKT4	Biolegend	317442	1 in 50
CXCR3	PE-CF594	1C6/CXCR3	BD Biosciences	562451	1 in 50
PD1	PE-Cy7	EH12.1	BD Biosciences	561272	1 in 50
Vd2	APC-Fire	B6	Biolegend	331420	1 in 50
Viability	LD-blue		Thermofisher	L23105	1 in 5000
**Intracellular:**					
IFNy	BUV395	B27	BD Biosciences	563563	1 in 50
IL-10	BV421	JES3-9D7	Biolegend	501422	1 in 10
TNF	BV750	MAb11	BD Biosciences	566359	1 in 100
IL-17a	FITC	BL168	Biolegend	512304	1 in 25
IL-21	PE	3A3-N2.1	BD Biosciences	560463	1 in 10
IL-4	APC	MP4-25D2	Biolegend	500812	1 in 10

Supplementary Table S5: Genes used in input for scType prediction *attached as excel*

Supplementary Table S6: Cluster markers for predicted annotated cell clusters in CHMI data set *attached as excel*

Supplementary Table S7: DEGs in each Tfh subset during malaria infection identified by edgeR pseudobulk *attached as excel*

Supplementary Table S8: Antibody panel for sort purification of Tfh cells for scRNAseq of healthy samples

Target	Fluorochrome	Clone	Manufacturer	Catalog #	Dilution
CD45RA	BB515	HI100	BD Biosciences	564552	1 in 400
CD4	PerCP-Cy5.5	OKT4	Biolegend	317428	1 in 50
CXCR3	PE-CF594	1C6/CXCR3	BD Biosciences	562451	1 in 50
PD1	PE-Cy7	EH12.1	BD Biosciences	561272	1 in 50
CD3	AF700	OKT3	Biolegend	317340	1 in 50
	Sytox Blue		Invitrogen	S34857	1 in 1000
CCR6	BV650	11A9	BD Biosciences	563922	1 in 50
CXCR5	BV711	J252D4	Biolegend	356933	1 in 50

Supplementary Table S9: Antibody panel for sort purification of Tfh cells for scRNAseq of CHMI samples

Target	Fluorochrome	Clone	Manufacturer	Catalog #	Dilution
**Stain 1:**					
TCR gD	FITC	B1	Biolegend	331208	3 in 25
CXCR5	BV711	J252D4	Biolegend	356933	2 in 25
**Stain 2:**					
CD4	PerCpCy5.5	OKT4	Biolegend	317428	1 in 50
CD19	PE	HIB19	BD Biosciences	555413	1 in 100
PD1	PE-Cy7	EH12.1	BD Biosciences	563922	1 in 50
Vd2	APC	B6	Biolegend	331418	1 in 50
CD3	AF700	OKT3	Biolegend	317340	1 in 50
	Sytox Blue		Invitrogen	S34857	1 in 1000
CD56	BV510	HCD56	Biolegend	318340	1 in 100
CD45RA	BV570	HI100	Biolegend	304132	1 in 400
HLADR	BV785	L243	Biolegend	307642	1 in 200

Supplementary Figures

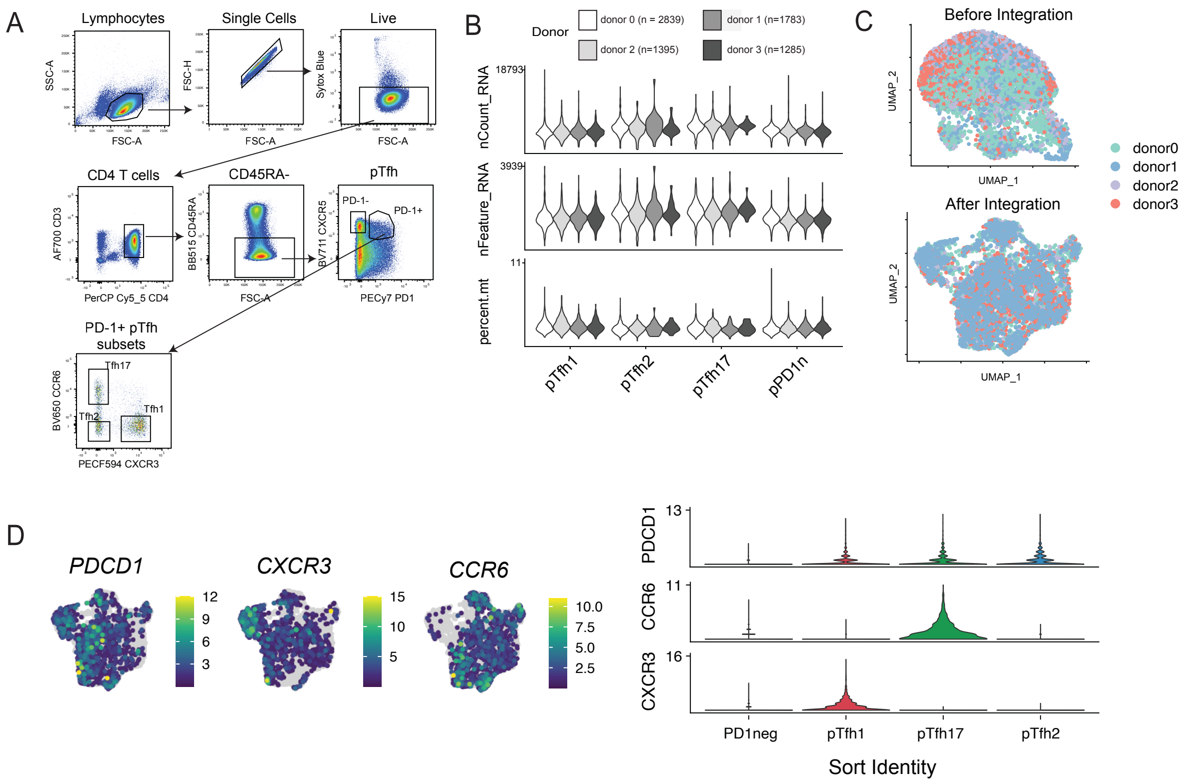

Supplementary Figure 1. Single cell RNA sequencing of pTfh cells from healthy donors.

A) Gating strategy for sorting pTfh cell populations scRNAseq. Non-naïve CD4 T cells were identified as CD3+/CD4+, CD45RA- cells. Tfh1 (CXCR3+CCR6-), Tfh2 (CXCR3-CCR6-), Tfh17(CXCR3-CCR6+) were sorted from CXCR5+PD1+ cells, and a resting Tfh cell population from CXCR5+PD1- cells. ***B)*** *QC of cells from each donor and subset, nCount_RNA, nFeature_RNA and percent mitochondrial content is shown.* ***C)*** *UMAP of data before and after integration for donor.* ***D)*** *Gene expression of key phenotyping markers PD1 (encoded by PDCD1), CXCR3 and CCR6 used to sort Tfh subsets phenotypically. UMAP and Vlnplot in each sorted population shown.*

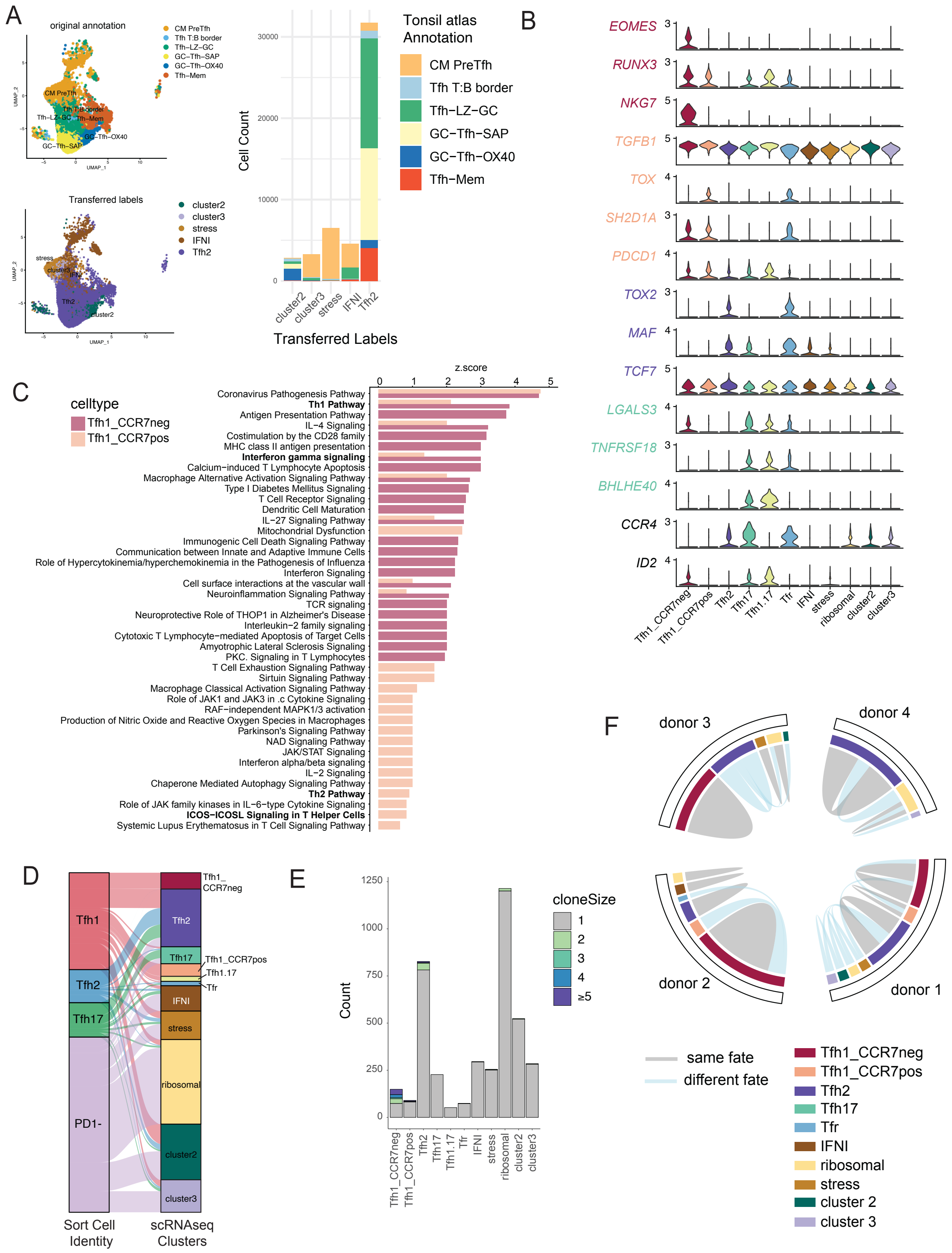

Supplementary Figure 2. Cluster identification in single cell RNA sequencing of pTfh cells from health donors

A) *UMAP and bar chart showing the number of cells in each predicted cell cluster grouped by their original cell annotation from the human tonsil atlas.* ***B)*** *Expression of genes with various roles in Tfh and CD4 T cell development across different Tfh cell clusters.* ***C)****. Ingenuity Pathway Analysis identified top 25 pathways from cluster marker genes (relative to all other Tfh clusters) for Tfh1-CCR7*^{neg} *and Tfh1-CCR7*^{pos} *subsets. Number of genes identified in enriched pathway indicated for*

each subset. Pathways of interest in bold. ***D)*** *Relationship between cell sorted identity and transitional cell cluster identify.* ***E)*** *Frequency of individual clones that occurred with clonal size of 1, 2, 3, 4, >5 in each subset.* ***F)*** *Circos plot of clonal overlap between subsets. Each cell of clones (≥2) is represented in a node in the ring, with a cell of each clone indicated by the inner coloured ring and donor by the outer labelled ring. Clones that exist in a single Tfh subset are joined by grey lines, while clones that are shared across Tfh subsets are joined by light blue lines. No clones were shared across donors.*

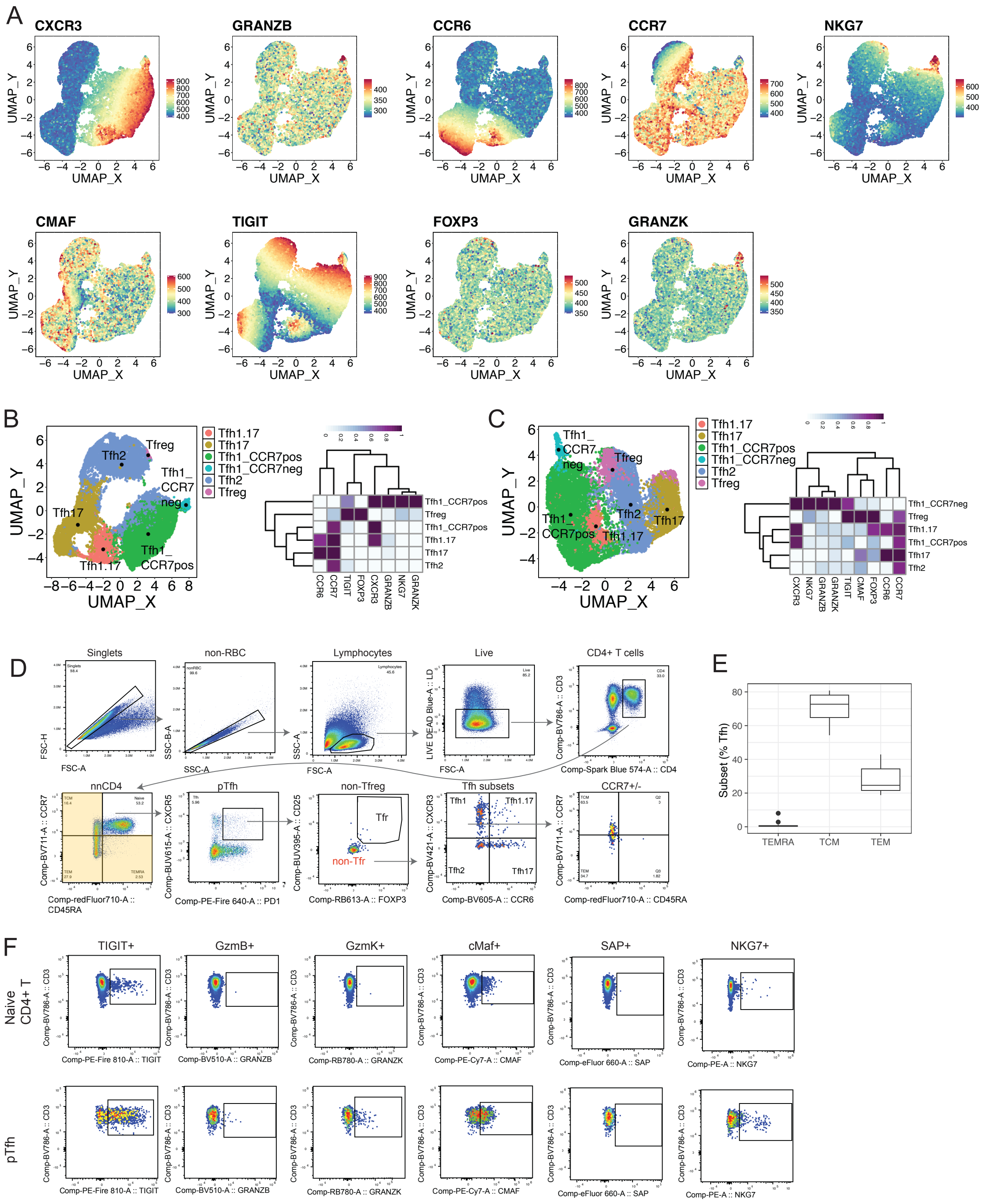

Supplementary Figure 3: Phenotypic diversity within pTfh subsets based on CCR7 expression.

A) *Expression of each marker used in unbiased analysis (see Figure 2).* ***B/C)*** *UMAP of unbias analysis of a single experiment (n=6 for B and n=6 for C).* ***D)*** *Gating strategy for identify pTfh cells. pTfh cells were identified as CXCR5+PD1+ non-naïve CD4 T cells. Within pTfh cells, Tfr cells were identified as FoxP3+/*$CD127^{lo}$ *cells, pTfh subsets identified based on CXCR3 and CCR6, and CCR7 expression analysed.* ***E)*** *Proportion of central memory (TCM, CCR7+CD45RA-), effector memory (TEM, CCR7+CD45RA-) and effector memory re-expressing CD45RA (TEMRA, CCR7-/CD45RA+) cells within Tfh cell population.* ***F)*** *Marker expression in naïve CD4 T cells and pTfh cells.*

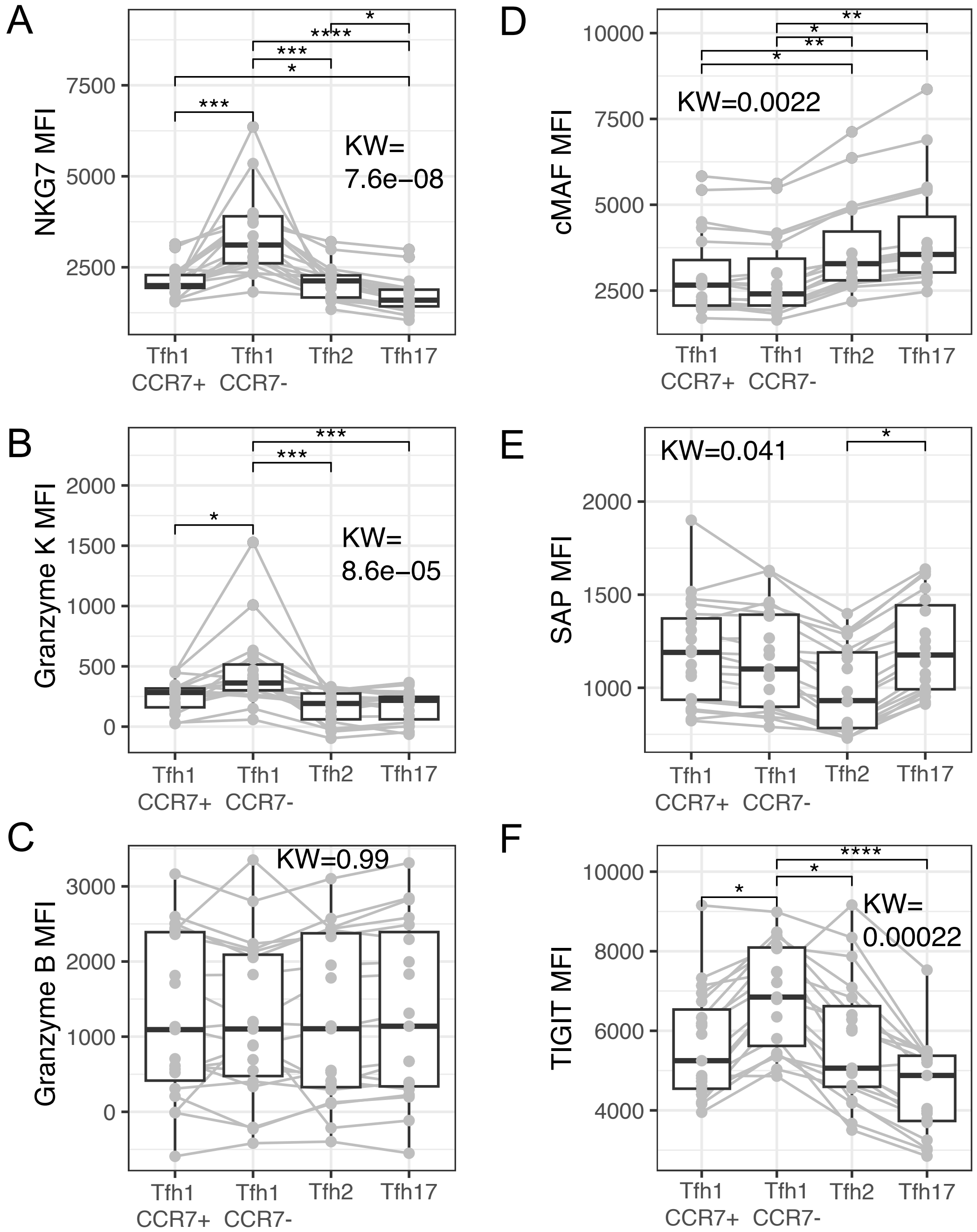

Supplementary Figure 4: Marker expression as MFI on Tfh subsets identified by flow cytometry.

*__A)__ NKG7, __B)__ granzyme K, __C)__ granzyme B, __D)__ cMAF, __E)__ SAP and __F)__ TIGIT MFI on each Tfh subset/phenotypic state in healthy individuals (n=19). Data are marker expression as MFI for each Tfh subset. Kruskal-Wallis test is used for the global comparison. P is post-hoc dunns test with FDR correction for multiple comparisons. Only significant differences (p<0.05) are shown. * <0.05-<0.01, ** <0.01-<0.001, ***<0.001.*

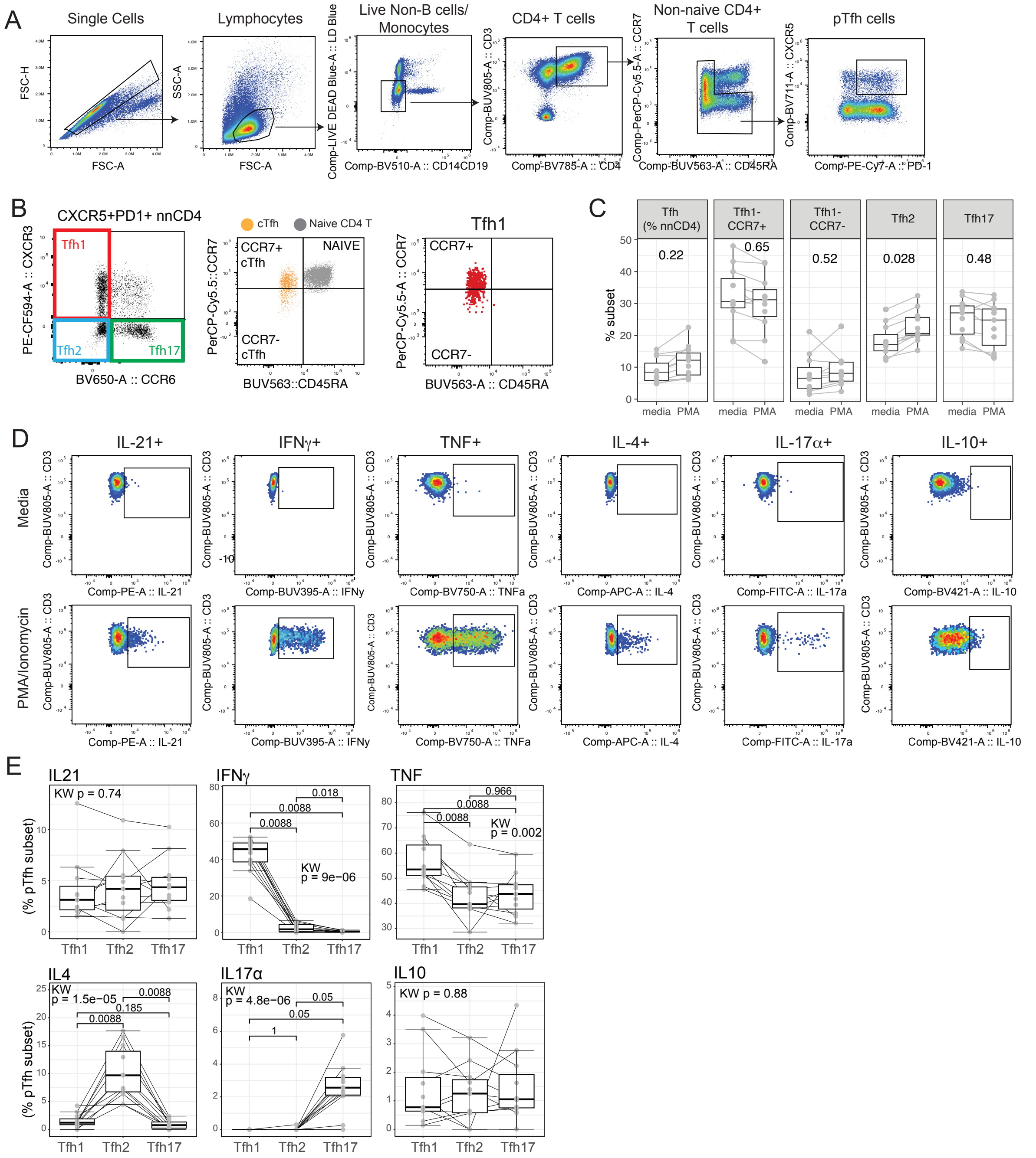

Supplementary Figure 5: Gating strategy for pTfh cells and cytokine expression following PMA/Io stimulation.

PBMCs were stimulated with PMA/Ionomycin to assess cytokine production. ***A)*** *pTfh cells were identified as CXCR5+PD1+ CD4 T cells.* ***B)*** *A gradient of CCR7 could be detected within pTfh1 cell susbsets, and pTfh1 clusters can be divided into CCR7^{pos} and CCR7^{neg} cells.* ***C)*** *Subset distribution before and after stimulation. Tfh is % of non-naïve CD4, while Tfh subsets are % of total Tfh. P is the paired Wilcoxon signed-rank test.* ***D)*** *Representative data of cytokine expression in pTfh cells in unstimulated (media) or PMA/Ionomycin stimulated cells.* ***E)*** *pTfh cells subsets were identified by CXCR3 and CCR6 expression as pTfh1 (CXCR3+CCR6+), pTfh2 (CXCR3-CCR6-) and pTfh17 (CXCR3-CCR6+) cells (grouping CCR7^{pos} and CCR7^{neg} Tfh1 cells). P is the paired Wilcoxon signed-rank test between groups, adjusted for multiple comparisons with Holm's FDR, and Kruskal Wallis group comparison test.*

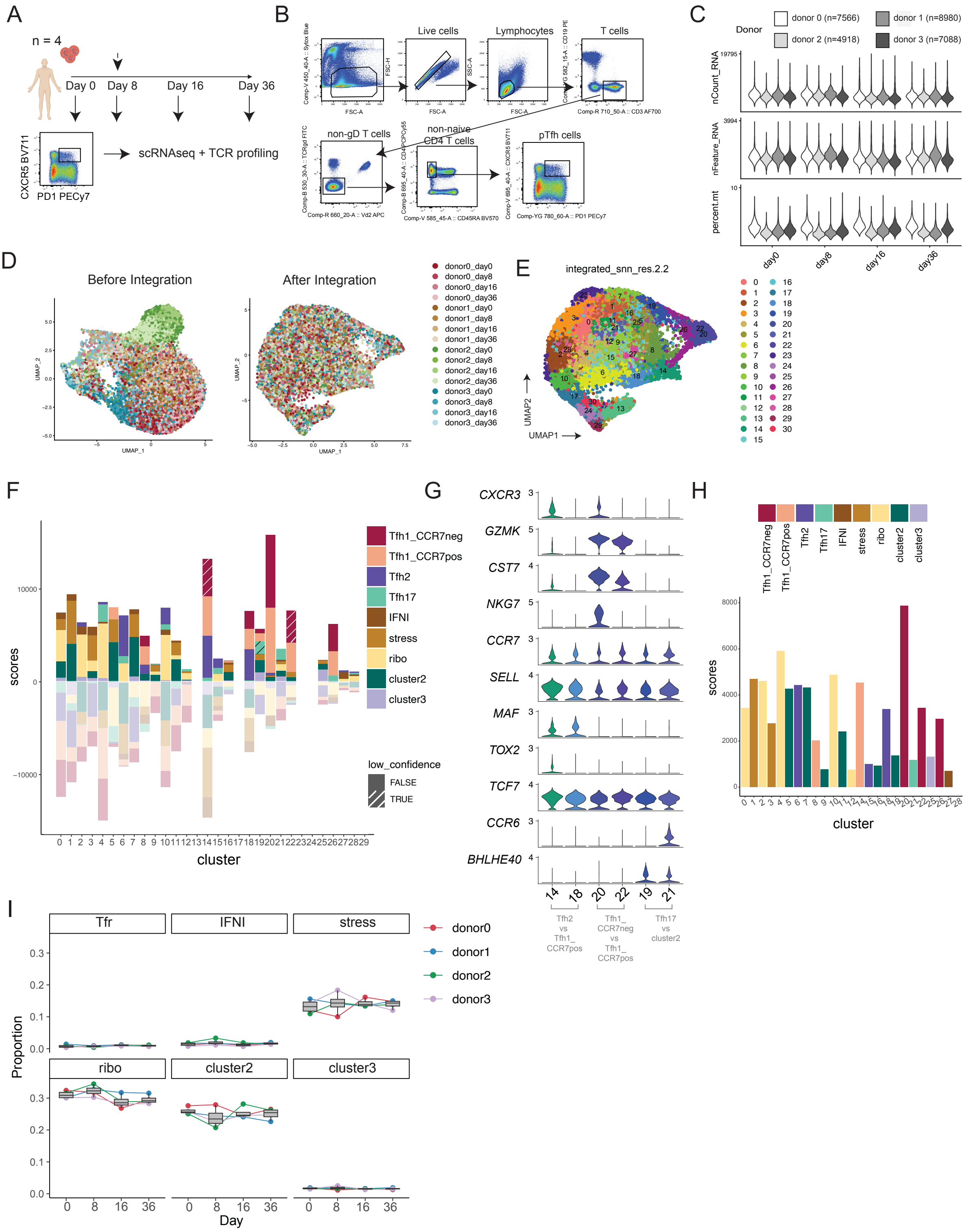

Supplementary Fig 6. scRNAseq analysis of Tfh cells in CHMI

A) *scRNAseq experimental set up for Tfh cells during controlled human malaria infection (CHMI). Tfh cells (CXCR5+PD1+) identified within non-naïve CD4 T cells were sorted from four individuals at day 0, 8, 16 and end-of-study during CHMI. Individuals were treated at day 8 (indicated by dotted arrow).* ***B)*** *Gating strategy for Tfh cell sorting for scRNAseq.* ***C)*** *QC of data from each donor across time points nCount_RNA, nFeature_RNA and percent mitochondrial content is shown.* ***D)*** *Data before and after integration based on donor and CHMI day.* ***E)*** *Over clustering of data prior to scType label*

transferer. ***F)*** *scType was used to label transfer cell signatures (top 20 up and down regulated genes) for each cell subset identified in healthy 'map' data onto over-clustered CHMI data. Each cell cluster was scored for each reference signature (left), and the majority score (right) used to collapse clusters into cell subsets.* ***G)*** *Average expression of Tfh cell cluster markers in each subset for manual adjustment of "low-confidence" predicted cluster identities.* ***H)*** *Final predicted label for each cell cluster.* ***I****) Proportion of other Tfh cell subsets identified over time.*

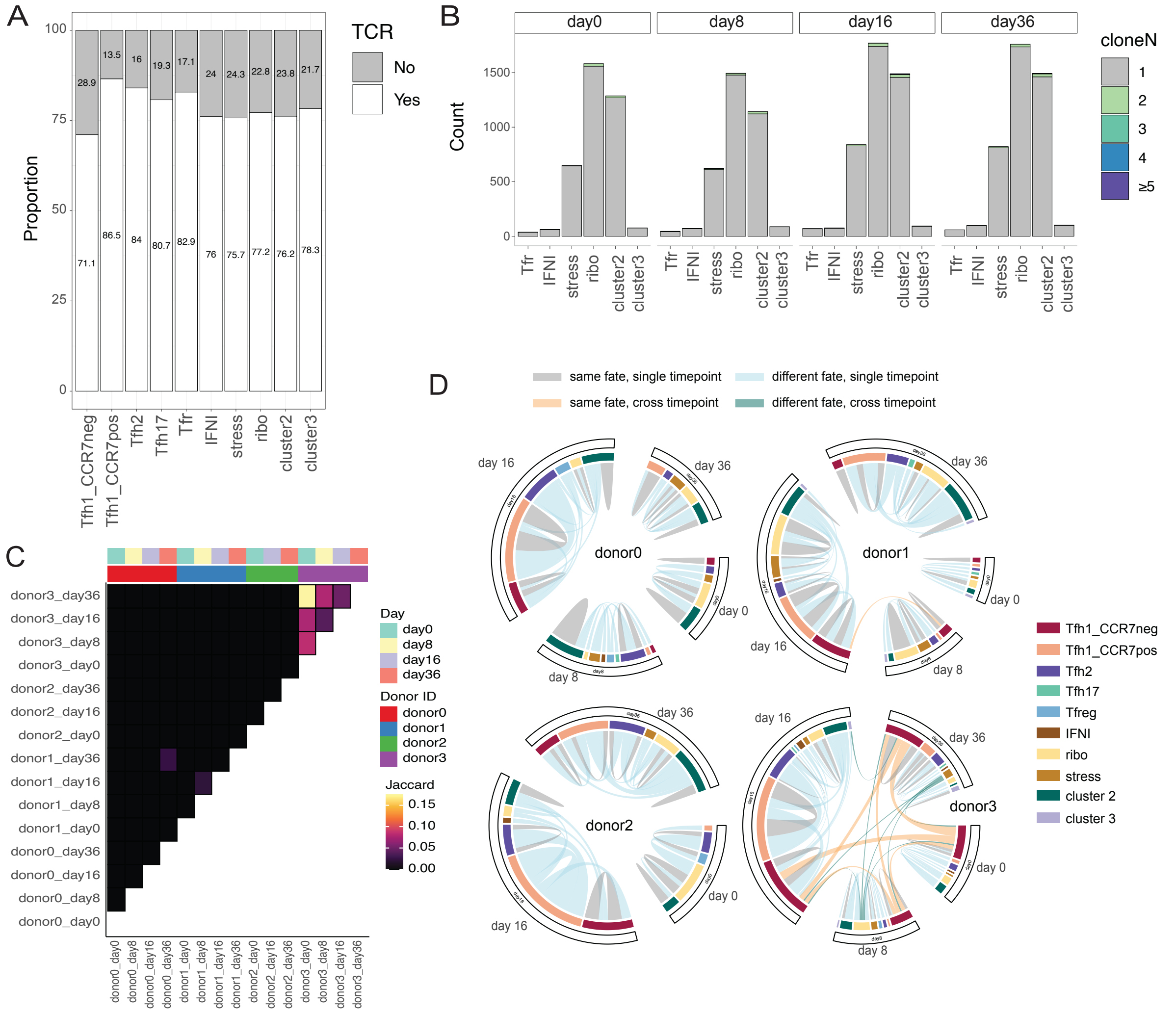

Supplementary Figure 7: TCR analysis of Tfh cells in CHMI

A) Proportion of cells with TCR captured across each subset. ***B****) Clonal size of other Tfh cell subsets identified over time.* ***C)*** *Clonal overlap between donor and day.* ***D)*** *Circos plot of clonal overlap between subsets for each donor. Each cell of clones (≥2) is represented in a node in the ring, with a cell of each clone indicated by the inner coloured ring and donor by the outer labelled ring. Clones that exist in a single Tfh subset are joined by grey lines, clones that exist in a single Tfh subset across more than one timepoint are in peach, clones that are shared across different Tfh subsets at a single timepoint are joined by light blue lines, and clones that are shared across different Tfh subsets and different timepoints are in green. No clones were shared across donors.*

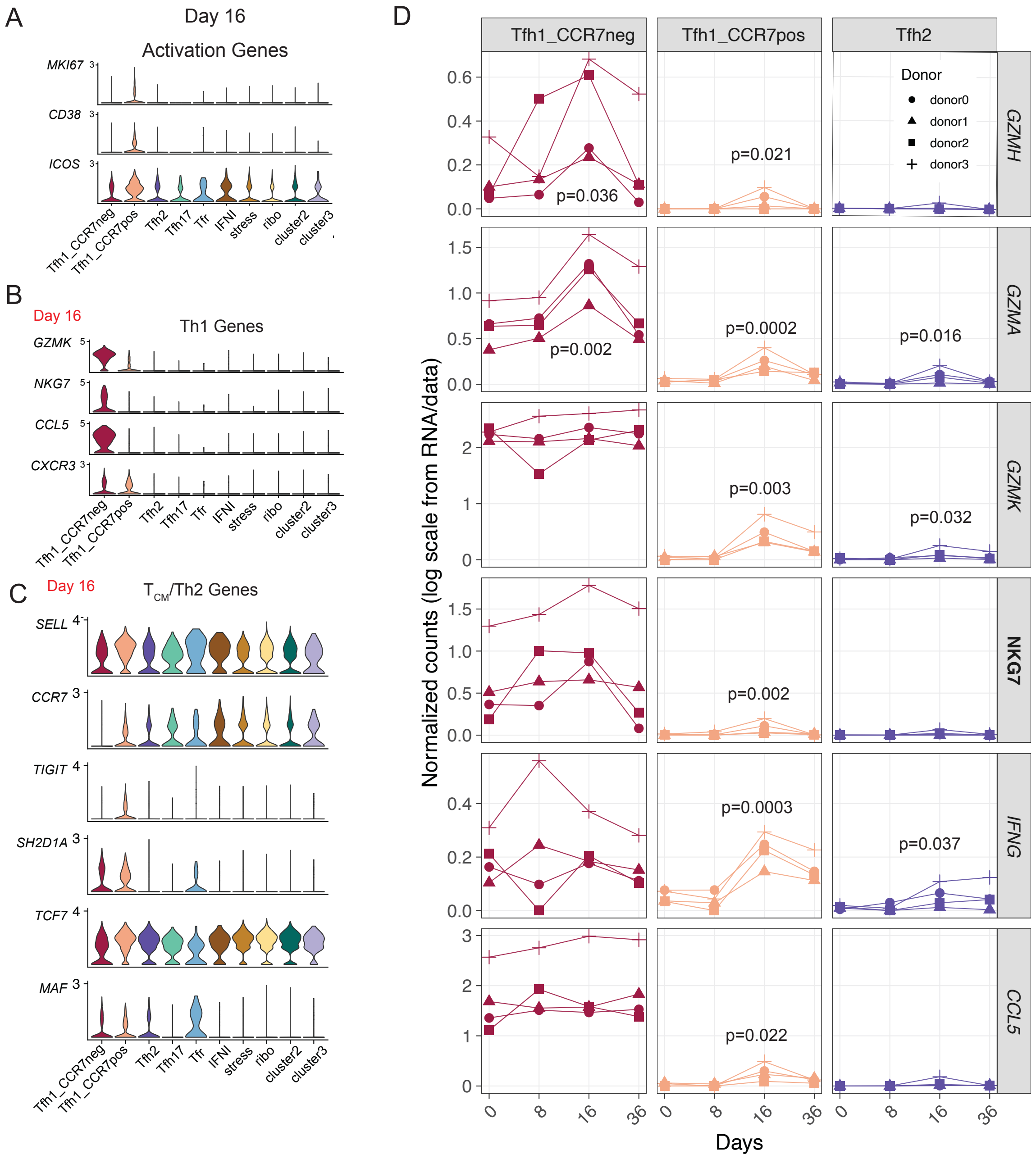

Supplementary Figure 8: Tfh cell activation profiles at day 16 following CHMI

DEGs were identified in each subsets comparing day 0 to subsequent time points during infection. ***A)*** *Expression of activation genes for all clusters at Day 16.* ***B-C)*** *Despite upregulation of genes associated with inflammation, expression profiles were still different between Tfh1_cyto and Tfh1_CCR7 subsets. Tfh cell subsets maintained expression profiles of identifying cluster markers.* ***D)*** *Expression of selected inflammatory and cytotoxic genes in each individual in each Tfh subset at day 0, 8, 16 and 36 of CHMI. P is adjusted from pseudobulk analysis at day 16 compared to Day 0.*

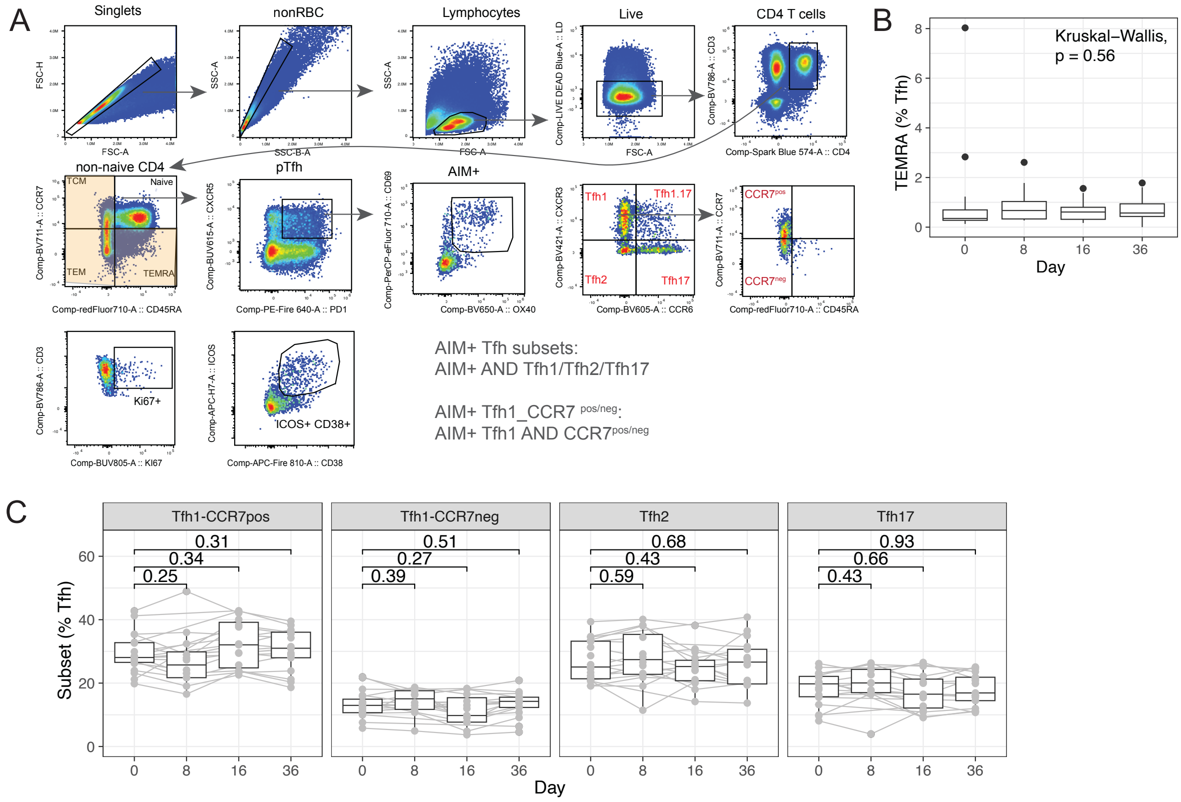

Supplementary Figure 9: Validating Tfh cell profiles in CHMI

A) *Gating strategy for identification of malaria specific pTfh cells in individuals undergoing CHMI using Activation Induced Marker assay.* ***B)*** *Proportion effector memory re-expressing CD45RA (TEMRA, CCR7-/CD45RA+) within total Tfh population across CHMI. Kruskal Wallis indicated.* ***C)*** *Frequencies of Tfh subsets within the total Tfh compartment across CHMI. P is Wilcoxon rank-sum test.*

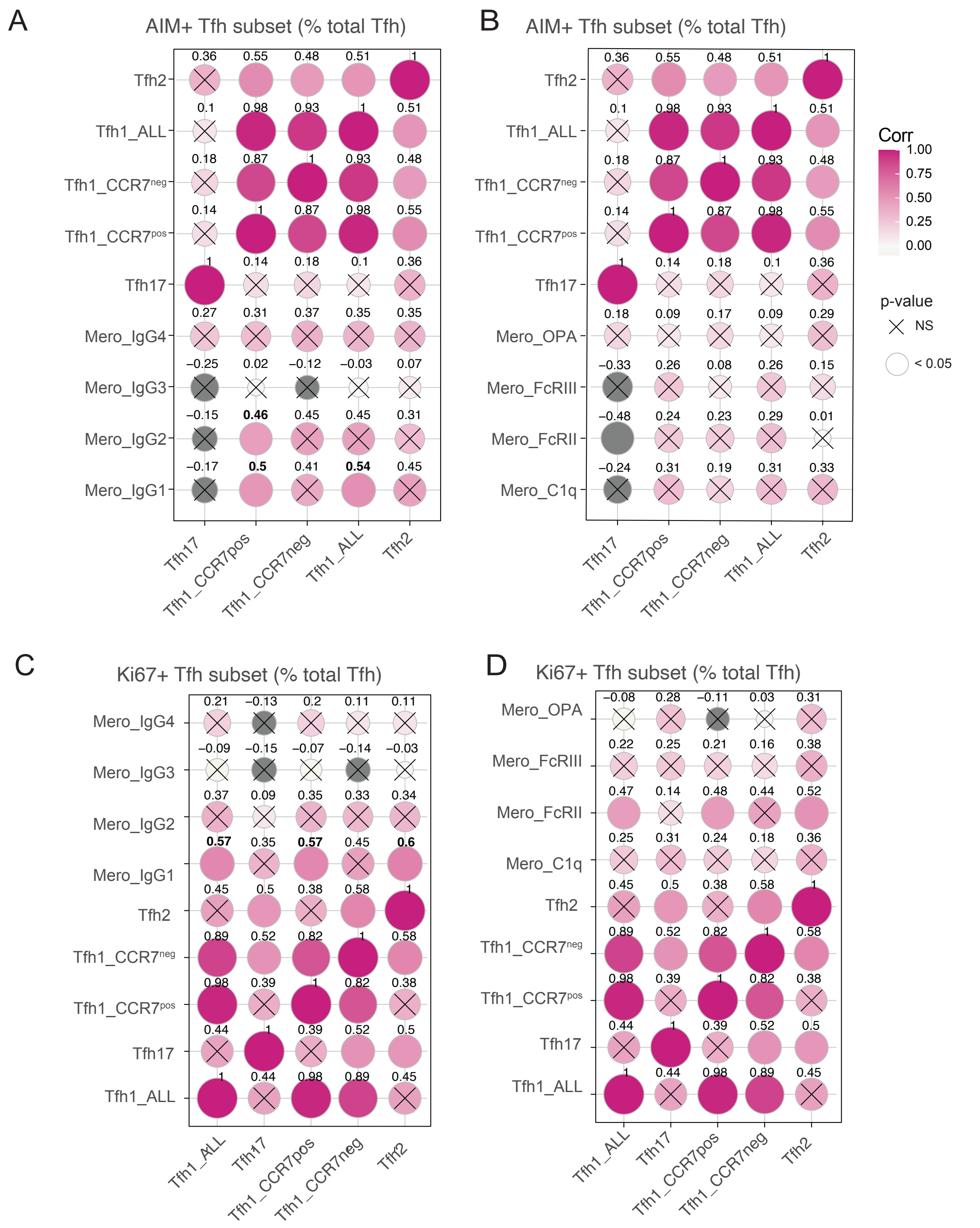

Supplementary Figure 10: Correlations between Tfh cells and antibody development in CHMI.

*Correlation matrix between malaria-specific Tfh subsets **(A/B)** or Ki67 expression of Tfh subsets **(C/D)** at day 36 with either IgG subclasses to merozoite **(A/C)** or functional capacity to fix complement (C1q), bind dimeric FcγRIIa or FcγRIIIa (surrogates for IgG antibody capacity to crosslink cellular receptors), and promote opsonic phagocytosis (OPA) **(B/C)**. Spearman's rho and p-value are indicated.*
